# An Empirical Comparison of Meta- and Mega-Analysis With Data From the ENIGMA Obsessive-Compulsive Disorder Working Group

**DOI:** 10.3389/fninf.2018.00102

**Published:** 2019-01-08

**Authors:** Premika S. W. Boedhoe, Martijn W. Heymans, Lianne Schmaal, Yoshinari Abe, Pino Alonso, Stephanie H. Ameis, Alan Anticevic, Paul D. Arnold, Marcelo C. Batistuzzo, Francesco Benedetti, Jan C. Beucke, Irene Bollettini, Anushree Bose, Silvia Brem, Anna Calvo, Rosa Calvo, Yuqi Cheng, Kang Ik K. Cho, Valentina Ciullo, Sara Dallaspezia, Damiaan Denys, Jamie D. Feusner, Kate D. Fitzgerald, Jean-Paul Fouche, Egill A. Fridgeirsson, Patricia Gruner, Gregory L. Hanna, Derrek P. Hibar, Marcelo Q. Hoexter, Hao Hu, Chaim Huyser, Neda Jahanshad, Anthony James, Norbert Kathmann, Christian Kaufmann, Kathrin Koch, Jun Soo Kwon, Luisa Lazaro, Christine Lochner, Rachel Marsh, Ignacio Martínez-Zalacaín, David Mataix-Cols, José M. Menchón, Luciano Minuzzi, Astrid Morer, Takashi Nakamae, Tomohiro Nakao, Janardhanan C. Narayanaswamy, Seiji Nishida, Erika L. Nurmi, Joseph O'Neill, John Piacentini, Fabrizio Piras, Federica Piras, Y. C. Janardhan Reddy, Tim J. Reess, Yuki Sakai, Joao R. Sato, H. Blair Simpson, Noam Soreni, Carles Soriano-Mas, Gianfranco Spalletta, Michael C. Stevens, Philip R. Szeszko, David F. Tolin, Guido A. van Wingen, Ganesan Venkatasubramanian, Susanne Walitza, Zhen Wang, Je-Yeon Yun, ENIGMA-OCD Working-Group, Paul M. Thompson, Dan J. Stein, Odile A. van den Heuvel, Jos W. R. Twisk

**Affiliations:** ^1^Department of Psychiatry, Amsterdam University Medical Centers (UMC), Vrije Universiteit Amsterdam, Amsterdam Neuroscience, Amsterdam, Netherlands; ^2^Department of Anatomy and Neurosciences, Amsterdam University Medical Centers, Vrije Universiteit Amsterdam, Amsterdam Neuroscience, Amsterdam, Netherlands; ^3^Department of Epidemiology and Biostatistics, Amsterdam Public Health Research Institute, Amsterdam University Medical Centers, Vrije Universiteit Amsterdam, Amsterdam, Netherlands; ^4^Orygen, The National Centre of Excellence in Youth Mental Health, Melbourne, VIC, Australia; ^5^Centre for Youth Mental Health, The University of Melbourne, Melbourne, VIC, Australia; ^6^Department of Psychiatry, Graduate School of Medical Science, Kyoto Prefectural University of Medicine, Kyoto, Japan; ^7^Department of Psychiatry, Bellvitge University Hospital, Bellvitge Biomedical Research Institute-IDIBELL, L'Hospitalet de Llobregat, Barcelona, Spain; ^8^Centro de Investigación Biomèdica en Red de Salud Mental (CIBERSAM), Barcelona, Spain; ^9^Department of Clinical Sciences, University of Barcelona, Barcelona, Spain; ^10^Department of Psychiatry, Faculty of Medicine, The Centre for Addiction and Mental Health, The Margaret and Wallace McCain Centre for Child, Youth and Family Mental Health, Campbell Family Mental Health Research Institute, University of Toronto, Toronto, ON, Canada; ^11^The Hospital for Sick Children, Centre for Brain and Mental Health, Toronto, ON, Canada; ^12^Department of Psychiatry, Yale University School of Medicine, New Haven, CT, United States; ^13^Mathison Centre for Mental Health Research and Education, Cumming School of Medicine, Hotchkiss Brain Institute, University of Calgary, Calgary, AB, Canada; ^14^Department of Psychiatry, Cumming School of Medicine, University of Calgary, Calgary, AB, Canada; ^15^Departamento de Psiquiatria, Faculdade de Medicina, Instituto de Psiquiatria, Universidade de São Paulo, São Paulo, Brazil; ^16^Division of Neuroscience, Psychiatry and Clinical Psychobiology, Scientific Institute Ospedale San Raffaele, Milan, Italy; ^17^Department of Psychology, Humboldt-Universität zu Berlin, Berlin, Germany; ^18^Obsessive-Compulsive Disorder (OCD) Clinic Department of Psychiatry National Institute of Mental Health and Neurosciences, Bangalore, India; ^19^Department of Child and Adolescent Psychiatry and Psychotherapy, Psychiatric Hospital, University of Zurich, Zurich, Switzerland; ^20^Magnetic Resonance Image Core Facility, IDIBAPS (Institut d'Investigacions Biomèdiques August Pi i Sunyer), Barcelona, Spain; ^21^Department of Child and Adolescent Psychiatry and Psychology, Hospital Clínic Universitari, Institute of Neurosciences, Barcelona, Spain; ^22^Department of Psychiatry, First Affiliated Hospital of Kunming Medical University, Kunming, China; ^23^Institute of Human Behavioral Medicine, SNU-MRC, Seoul, South Korea; ^24^Laboratory of Neuropsychiatry, Department of Clinical and Behavioral Neurology, IRCCS Santa Lucia Foundation, Rome, Italy; ^25^Neurosciences, Psychology, Drug Research and Child Health (NEUROFARBA), University of Florence, Florence, Italy; ^26^Department of Psychiatry, Amsterdam Neuroscience, Amsterdam University Medical Centers, University of Amsterdam, Amsterdam, Netherlands; ^27^Netherlands Institute for Neuroscience, Royal Netherlands Academy of Arts and Sciences, Amsterdam, Netherlands; ^28^Department of Psychiatry and Biobehavioral Sciences, University of California, Los Angeles, Los Angeles, CA, United States; ^29^Department of Psychiatry, University of Michigan, Ann Arbor, MI, United States; ^30^MRC Unit on Risk & Resilience in Mental Disorders, Department of Psychiatry, University of Cape Town, Cape Town, South Africa; ^31^Imaging Genetics Center, Keck School of Medicine of the University of Southern California, Mark and Mary Stevens Neuroimaging and Informatics Institute, Marina del Rey, CA, United States; ^32^Shanghai Mental Health Center Shanghai Jiao Tong University School of Medicine, Shanghai, China; ^33^De Bascule, Academic Center for Child and Adolescent Psychiatry, Amsterdam, Netherlands; ^34^Department of Child and Adolescent Psychiatry, Amsterdam University Medical Centers, University of Amsterdam, Amsterdam, Netherlands; ^35^Yeongeon Student Support Center, Seoul National University College of Medicine, Seoul, South Korea; ^36^Department of Psychiatry, Oxford University, Oxford, United Kingdom; ^37^Department of Neuroradiology, Klinikum rechts der Isar, Technische Universität München, Munich, Germany; ^38^TUM-Neuroimaging Center (TUM-NIC) of Klinikum rechts der Isar, Technische Universität München, Munich, Germany; ^39^Department of Psychiatry, Seoul National University College of Medicine, Seoul, South Korea; ^40^Department of Brain and Cognitive Sciences, Seoul National University College of Natural Sciences, Seoul, South Korea; ^41^Institut d'Investigacions Biomèdiques August Pi i Sunyer (IDIBAPS), Barcelona, Spain; ^42^Department of Medicine, University of Barcelona, Barcelona, Spain; ^43^SU/UCT MRC Unit on Anxiety and Stress Disorders, Department of Psychiatry, University of Stellenbosch, Stellenbosch, South Africa; ^44^Columbia University Medical College, Columbia University, New York, NY, United States; ^45^The New York State Psychiatric Institute, New York, NY, United States; ^46^Department of Clinical Neuroscience, Centre for Psychiatry Research, Karolinska Institutet, Stockholm, Sweden; ^47^Mood Disorders Clinic, St. Joseph's HealthCare, Hamilton, ON, Canada; ^48^Department of Neuropsychiatry, Graduate School of Medical Sciences, Kyushu University, Fukuoka, Japan; ^49^ATR Brain Information Communication Research Laboratory Group, Kyoto, Japan; ^50^Center for Mathematics, Computing and Cognition, Universidade Federal do ABC, Santo Andre, Brazil; ^51^Center for OCD and Related Disorders, New York State Psychiatric Institute, New York, NY, United States; ^52^Anxiety Treatment and Research Center, St. Joseph's HealthCare, Hamilton, ON, Canada; ^53^Department of Psychobiology and Methodology of Health Sciences, Universitat Autònoma de Barcelona, Barcelona, Spain; ^54^Beth K. and Stuart C. Yudofsky Division of Neuropsychiatry, Department of Psychiatry and Behavioral Sciences, Baylor College of Medicine, Houston, TX, United States; ^55^Yale University School of Medicine, New Haven, CT, United States; ^56^Clinical Neuroscience and Development Laboratory, Olin Neuropsychiatry Research Center, Hartford, CT, United States; ^57^Icahn School of Medicine at Mount Sinai, New York, NY, United States; ^58^James J. Peters VA Medical Center, Bronx, NY, United States; ^59^Institute of Living/Hartford Hospital, Hartford, CT, United States; ^60^Shanghai Key Laboratory of Psychotic Disorders, Shanghai, China

**Keywords:** neuroimaging, MRI, IPD meta-analysis, mega-analysis, linear mixed-effect models

## Abstract

**Objective:** Brain imaging communities focusing on different diseases have increasingly started to collaborate and to pool data to perform well-powered meta- and mega-analyses. Some methodologists claim that a one-stage individual-participant data (IPD) mega-analysis can be superior to a two-stage aggregated data meta-analysis, since more detailed computations can be performed in a mega-analysis. Before definitive conclusions regarding the performance of either method can be drawn, it is necessary to critically evaluate the methodology of, and results obtained by, meta- and mega-analyses.

**Methods:** Here, we compare the inverse variance weighted random-effect meta-analysis model with a multiple linear regression mega-analysis model, as well as with a linear mixed-effects random-intercept mega-analysis model, using data from 38 cohorts including 3,665 participants of the ENIGMA-OCD consortium. We assessed the effect sizes and standard errors, and the fit of the models, to evaluate the performance of the different methods.

**Results:** The mega-analytical models showed lower standard errors and narrower confidence intervals than the meta-analysis. Similar standard errors and confidence intervals were found for the linear regression and linear mixed-effects random-intercept models. Moreover, the linear mixed-effects random-intercept models showed better fit indices compared to linear regression mega-analytical models.

**Conclusions:** Our findings indicate that results obtained by meta- and mega-analysis differ, in favor of the latter. In multi-center studies with a moderate amount of variation between cohorts, a linear mixed-effects random-intercept mega-analytical framework appears to be the better approach to investigate structural neuroimaging data.

## Introduction

Data pooling across individual studies has the potential to significantly accelerate progress in brain imaging (Van Horn et al., 2001), as demonstrated by large-scale neuroimaging initiatives, such as the ENIGMA (Enhanced NeuroImaging Genetics through Meta-Analysis) consortium (Thompson et al., 2014). The most immediate advantage of data pooling is increased power due to the larger number of subjects available for analysis. Data pooling across multiple centers worldwide can also lead to a more heterogeneous and potentially representative participant sample. Large-scale studies are well-powered to distinguish consistent, generalizable findings from false positives that emerge from smaller-sampled studies. The participation of many experts may also lead to a more balanced interpretation, wider endorsement of the conclusions by others, and greater dissemination of results (Stewart, 1995).

An aggregate data meta-analysis is the most conventional approach, where summary results, such as effect size estimates, standard errors, and confidence intervals, are extracted from primary published studies and then synthesized to estimate the overall effect for all the studies combined (de Bakker et al., 2008). This approach is relatively quick and inexpensive, but often prone to selective reporting in primary studies, publication bias, low power to detect interaction effects and lack of harmonization of data processing and analysis methods among the included studies. To overcome these issues, collaborative groups are increasingly collating individual-participant data (IPD) from multiple studies to jointly analyze the individual-level data in a meta-analysis of IPD (Stewart, 1995). The IPD approach allows standardization of processing protocols and statistical analyses, culminating in study results not provided by the individual publications. This approach also allows modeling of interaction effects within the studies. Given these advantages, the IPD approach is currently the gold standard.

There are two competing statistical approaches for IPD meta-analysis: a two-stage or a one-stage approach (Thomas et al., 2014). In the two-stage approach, the first step includes analyzing the IPD from each study separately, to obtain aggregate (summary) data (e.g., effect size estimates and confidence intervals). The second step includes using standard meta-analytical techniques, such as a random effects meta-analysis model. The alternative one-stage approach analyzes all IPD in one statistical model while accounting for clustering among patients in the same study, to estimate an overall effect. Throughout this manuscript, the one-stage IPD approach is referred to as *mega-analysis*, while the two-stage approach is referred to as *meta-analysis*.

Some methodologists claim that a mega-analysis can be superior to meta-analysis. The comprehensive evaluation of missing data and greater flexibility in the control of confounders at the level of individual patients and specific studies are significant advantages of a mega-analytical approach. Mega-analyses have also been recommended as they avoid the assumptions of within-study normality and known within-study variances, which are especially problematic with smaller samples (Debray et al., 2013). Despite these advantages, mega-analysis requires homogeneous data sets and the establishment of a common centralized database. The latter criterion is time-consuming since cleaning, checking, and re-formatting the various data sets adds to the time and costs of performing mega-analyses. Obtaining IPD may also be challenging and limited by the terms of the informed consent or other data sharing constraints within each study. These are the main reasons why researchers often prefer meta-analysis using summary statistics. Additionally, meta-analysis allows for analyses of individual studies to account for local population substructure and study-specific covariates that may be better dealt with within each study. While each method has its own advantages and limitations, researchers still debate which method is superior for tackling different types of questions [see (Stewart and Tierney, 2002; Burke et al., 2017) for reviews on advantages and disadvantages of each approach].

Brain imaging communities focusing on different diseases have started collaborating to perform well-powered meta- and mega-analyses. In the largest studies to date on the neural correlates of OCD, the authors of the ENIGMA-OCD consortium (Boedhoe et al., 2017a, 2018) conducted a mega-analysis, pooling individual participant-level data from more than 25 research institutes worldwide, as well as a meta-analysis by combining summary statistic results from the independent sites. The meta- and mega-analyses revealed comparable findings of subcortical abnormalities in OCD (Boedhoe et al., 2017a), but the mega-analytical approach seemed more sensitive for detecting subtle cortical abnormalities (Boedhoe et al., 2018). Before definitive conclusions regarding the performance of either method can be drawn, it is necessary to critically evaluate the results obtained by various approaches for meta- and mega-analyses.

Herein, we use data from the ENIGMA-OCD consortium to compare results obtained by meta- and mega-analyses. Specifically, we applied the inverse variance weighted random-effect meta-analysis model and the multiple linear regression mega-analysis model as used in the aforementioned studies (Boedhoe et al., 2017a, 2018). In addition, we compared findings from these models to those detected with a linear mixed-effects random-intercept mega-analytical model. Effect sizes and standard error estimates, and (where possible) model fit were used to evaluate which of the methods performs best.

## Methods

### Samples

The ENIGMA-OCD working group includes 38 data sets from 27 international research institutes with neuroimaging and clinical data from OCD patients and typically developing healthy control subjects, including both children and adults (Boedhoe et al., 2018). We defined adults as individuals aged ≥18 years and children as individuals aged < 18 years. The split at the age of 18 followed from a natural selection of the age ranges used in these samples, as most samples used the age of 18 years as a cut-off for inclusion. Because our previous findings and the literature suggest differential effects between pediatric and adult samples, we performed separate analyses for adult and pediatric data [for demographics and further details on the samples, see (Boedhoe et al., 2018)]. In total, we analyzed data from 3,665 participants including 1,905 OCD patients (407 children and 1,498 adults) and 1,760 control participants (324 children and 1,436 adults). All local institutional review boards permitted the use of measures extracted from the coded data for analyses.

### Image Acquisition and Processing

Structural T1-weighted brain MRI scans were acquired and processed locally. For image acquisition parameters of each site, please see (Boedhoe et al., 2018). All cortical parcellations were performed with the fully automated segmentation software FreeSurfer, version 5.3 (Fischl, 2012), following standardized ENIGMA protocols to harmonize analyses and quality control procedures across multiple sites (see http://enigma.usc.edu/protocols/imaging-protocols/). Segmentations of 68 (34 left and 34 right) cortical gray matter regions based on the Desikan-Killiany atlas (Desikan et al., 2006) and two whole-hemisphere measures were visually inspected and statistically evaluated for outliers [see (Boedhoe et al., 2018) for further details on quality checking].

### Statistical Framework

We examined differences between OCD patients and controls across samples by performing (1) an inverse variance weighted random-effects meta-analysis model; (2) a multiple linear regression mega-analysis model; and (3) a linear mixed-effects random-intercept mega-analysis model. Each of the 70 cortical regions of interest (68 regions and two whole-hemisphere averages) served as the outcome measure and a binary indicator of diagnosis as the predictor of interest. In the meta-analysis, all cortical thickness models were adjusted for age and sex (Im et al., 2008; Westlye et al., 2010), and all cortical surface area models were corrected for age, sex, and intracranial volume (Barnes et al., 2010; Ikram et al., 2012). In the mega-analysis all models were also adjusted for scanning center (cohort). The two mega-analytical frameworks are similar, but the models account differently for clustering of data within cohorts; linear regression with a dummy variable for each cohort and linear mixed-effects models (more efficiently) with only one variance parameter. Finally, all models were fit using the restricted maximum likelihood method [REML (Harville, 1977)].

The meta- and mega-analysis encompass intrinsically different statistics, including differences in approaches for dealing with missing data. E.g., the mega-analysis estimates one restricted maximum likelihood over the entire data set. This estimation contains information of each of the other cohorts. The first stage of the meta-analysis includes the estimation of a restricted maximum likelihood per cohort, making this method more vulnerable to missing outcome data. Therefore, we descriptively compared the meta- and mega-analyses by examining the confidence intervals and standard error estimates for the effect sizes assessed. In addition, the Bayesian information criterion (BIC) were used to evaluate which of the mega-analytical models performs better. A lower BIC indicates a better model fit. Throughout the manuscript, we report *p* < 0.001.

### Meta-Analysis

We analyzed the IPD from each study to obtain aggregated summary data. Effect size estimates were calculated using Cohen's *d*, computed from the t-statistic of the diagnosis indicator variable from the regression models [(Nakagawa and Cuthill, 2007), *equation 10*]. All regression models and effect size estimates were fitted at each site separately. A final Cohen's *d* effect size estimate was obtained using an inverse variance-weighted random-effect meta-analysis model in R (metafor package, version 1.9-118). This meta-analytic framework enabled us to combine data from multiple sites and take the sample size of each cohort into account by weighing individual effect size estimates for the inverse variance per cohort.

### Mega-Analysis

We pooled all IPD in one statistical model to perform mega-analyses and fitted the following models:

#### Linear Regression

The linear regression model included cohorts as dummy variables. Effect size estimates were calculated using the Cohen's *d* metric computed from the t-statistic of the diagnosis indicator variable from the regression models [(Nakagawa and Cuthill, 2007), *equation 10*].

#### Linear Mixed-Effects Model – Random-Intercept

Linear mixed-effects models are extensions of linear regression models and efficiently account for clustering of data within cohorts. By adding a random-intercept for cohort, the adjustment for the clustering of data within cohorts is performed with only one (variance) parameter, which reduces the number of estimated parameters (rather than estimating the intercept of each dummy variable separately as in the linear regression model described above). We used *lme4* (linear mixed-effects analysis) package in R to perform the analyses. Effect size estimates were calculated using the Cohen's *d* metric computed from the *t*-values from the mixed-effects model [(Nakagawa and Cuthill, 2007), *equation 22*].

## Results

The results of the meta-analysis and linear regression mega-analysis have been published previously (Boedhoe et al., 2018). In this paper, we added the linear mixed-effects random-intercept mega-analysis and statistically compared the various approaches.

### Meta-Analysis

No significant differences (*p* < 0.001) in cortical thickness were observed in adult OCD patients (*N* = 1,498) compared to healthy controls (*N* = 1,436) (Supplementary Table S1). The meta-analysis did reveal a lower surface area of the transverse temporal cortex (Cohen's *d* −0.17) in OCD patients (Supplementary Table S2). No group differences in cortical thickness or surface area were observed in children with OCD (*N* = 407) compared to control children (*N* = 324) (Supplementary Tables S3, S4).

### Mega-Analysis

Both the linear regression (Cohen's *d* −0.14) and the linear mixed-effects random-intercept (Cohen's *d* −0.11) models revealed significantly lower cortical thickness in bilateral inferior parietal cortices in adult OCD patients (*N* = 1,498) compared to healthy controls (*N* = 1,436) (Supplementary Table S5). Both models also showed significantly lower surface area (Cohen's *d* −0.16) in the left transverse temporal cortex in OCD patients (Supplementary Table S6).

Both the linear regression (Cohen's *d* between −0.24 and −0.31) and the linear mixed-effects random-intercept (Cohen's *d* between −0.20 and −0.28) models revealed significantly thinner cortices in pediatric OCD patients (*N* = 407) compared with control children (*N* = 324) in the right superior parietal, left inferior parietal, and left lateral occipital cortices (Supplementary Tables S7). Neither model revealed significant group differences in cortical surface area (Supplementary Tables S8).

### Comparing Meta- and Mega-Analysis

#### Effect Sizes

When looking at the magnitude and order of effect sizes we see the same pattern resulting from the meta-analysis and linear regression mega-analysis in both the pediatric (Supplementary Tables S3, S7) and adult (Supplementary Tables S1, S5) datasets, i.e., the magnitude and direction of effect of the effect sizes derived from the meta-analysis and linear regression mega-analysis were highly similar. The linear mixed-effects random-intercept mega-analysis also showed a similar pattern of results, but slightly smaller effect sizes (Table 1 and Supplementary Tables S5, S7).

**Table 1:** Effect size, confidence interval, and standard error estimates, and BIC values of the main findings.

			**Cohen's d**	**Standard Error**	**95% CI**	**BIC**
**ADULT**						
Left inferior parietal cortex		meta-analysis	−0.13	0.053	−0.237 to −0.029	–
		mega-analysis LR^*^	−0.14	0.038	−0.211 to −0.063	9,693
	cortical thickness	mega-analysis LMEri^*^	−0.11	0.038	−0.214 to −0.065	9,638
Right inferior parietal cortex		meta-analysis	−0.13	0.046	−0.221 to −0.042	–
		mega-analysis LR^*^	−0.14	0.038	−0.211 to −0.064	9,799
		mega-analysis LMEri^*^	−0.10	0.038	−0.213 to −0.066	9,750
Left transverse temporal cortex	cortical surface area	meta-analysis^*^	−0.17	0.038	−0.243 to −0.095	–
		mega-analysis LR^*^	−0.16	0.037	−0.238 to −0.092	33,368
		mega-analysis LMEri^*^	−0.16	0.037	−0.240 to −0.095	33,263
**PEDIATRIC**						
Right superior parietal cortex		meta-analysis	−0.27	0.138	−0.540 to −0.001	–
		mega-analysis LR^*^	−0.27	0.075	−0.416 to −0.121	2,645
		mega-analysis LMEri^*^	−0.21	0.075	−0.415 to −0.119	2,634
Left inferior parietal cortex	cortical thickness	meta-analysis	−0.31	0.144	−0.593 to −0.027	–
		mega-analysis LR^*^	−0.31	0.077	−0.457 to −0.154	2,634
		mega-analysis LMEri^*^	−0.28	0.077	−0.455 to −0.151	2,610
Left lateral occipital cortex		meta-analysis	−0.26	0.095	−0.445 to −0.071	–
		mega-analysis LR^*^	−0.26	0.075	−0.404 to −0.109	2,464
		mega-analysis LMEri^*^	−0.23	0.075	−0.401 to −0.106	2,444

*LR, linear regression; LMEri, linear mixed-effects random-intercept model; CI, confidence interval; BIC = Bayesian information criterion*.

* *Indicates significant group difference at a threshold of p < 0.001*.

#### Standard Error and 95% Confidence Intervals

Overall, linear regression and linear mixed-effects random-intercept models showed lower standard errors and narrower confidence intervals than the meta-analysis. Similar standard errors and confidence intervals were found for the different mega-analysis models (Table 1 and Supplementary Tables S1–S8).

#### Goodness-of-Fit

The linear mixed-effects random-intercept models showed lower BIC values compared to the linear regression mega-analysis (Table 1 and Supplementary Tables S9–S12).

## Discussion

The aim of this study was to evaluate different statistical methods for large-scale multi-center neuroimaging analyses. We empirically evaluated whether a meta-analysis provides results comparable to a mega-analysis and which analytical framework performs better. Clinical interpretation of the results can be found elsewhere (Boedhoe et al., 2017b, 2018). Although effect sizes were similar for the meta-analysis and linear regression mega-analysis, lower standard errors and narrower confidence intervals of both mega-analytical approaches compared to the meta-analysis suggest better performance of the mega-analytical approach over the meta-analytical approach. While the meta-analysis failed to detect cortical thickness differences in both the adult and pediatric samples, it did support the findings of the mega-analyses at a less stringent significance threshold (*p* < 0.05 uncorrected). As a second aim, we investigated which mega-analytical framework was superior. The BIC values indicated a better model fit of the linear mixed-effects random-intercept model compared to the linear regression mega-analytical model.

Whereas, the linear regression model showed similar standard errors and confidence intervals to the linear mixed-effects random-intercept model, the latter fitted the data better. The effect sizes of the linear regression model appeared to be higher than those of the linear mixed-effects models, possibly indicating an overestimation of the effect of diagnosis. Indeed fixed-effects analyses (comparable to the linear regression models in our case) are reported to produce biased estimates or inflated type I error rates when pooled data includes cohorts with a small number of patients (Agresti and Hartzel, 2000; Kahan and Morris, 2012). Mathew and Nordstorm (2010) also suggested that a mega-analysis (one-stage approach) with a random intercept term might be slightly more precise than a meta-analysis (two-stage approach), which has a distinct intercept term per study (Mathew and Nordstorm, 2010). Taken together, our results suggest that the linear mixed-effects random-intercept mega-analysis model is the better approach for analyzing cortical gray matter data in a multi-center neuroimaging study.

We also explored (data not shown) a linear mixed-effects random-intercept and random-slope mega-analytical approach, since the various cohorts might have shown differences in effects of diagnosis related to clinical heterogeneity between patient samples. However, for most of the regions of interest the model did not converge. These computational difficulties and convergence problems have been reported before (Debray et al., 2013). As a result, effect sizes, confidence intervals, standard errors, and BIC values could not be estimated accurately. Indeed previous literature has demonstrated that mega-analyses may produce downwardly biased coefficient estimates when an incorrect model is specified, for instance when random effects are wrongly assumed (Dutton, 2010). Note that including a random slope in the linear mixed-effects model might be valuable when large variance is present in the data between cohorts. Therefore, we recommend the following strategy: (1) run a mixed-effects model with a random-intercept to correct for clustering of participants within cohorts; (2) add a random-slope to correct for potential variance in effects between cohorts; (3) and perform a likelihood-ratio test to statistically compare both models. If the likelihood-ratio test is significant i.e., there is a better fit of the random-intercept random-slope model, this model is preferred over the random-intercept only model. If the likelihood-ratio test is not significant i.e., there is a better fit of the random-intercept only model, this model is preferred over the random-intercept random-slope model.

Olkin and Sampson (1998) showed that for comparing treatments with respect to a continuous outcome in clinical trials, meta-analysis is equivalent to mega-analysis if the treatment effects and error variances are constant across trials. The equivalence has been extended even if the error variances are different across trials (Mathew and Nordstrom, 1999). Lin and Zeng theoretically and empirically showed asymptotic equivalence between meta- and mega-analyses when the effect sizes are the same for all studies (Lin and Zeng, 2010a,b). The different cohorts in our study did not all show similar effect sizes and error variances, possibly explaining why we did not find the meta- and mega-analyses to be equivalent. In practice, effect sizes and error variances vary across studies more often than not. Moreover, these authors (Lin and Zeng, 2010a) focused on a fixed-effects meta-analysis rather than a random-effects meta-analysis which is carried out in the current study. A fixed-effect model only takes into account the random error within cohorts, whereas the random-effect model also takes into account the random error between cohorts (Borenstein et al., 2010). Not taking into account the random error between different cohorts in neuroimaging data, for example, may lead to potentially misleading conclusions. More comprehensive simulation studies may be performed to assess theoretical differences in the results of meta- and mega-analyses. Such simulation studies covering various scenarios regarding varying effect sizes and error variances would strengthen our findings.

Conclusions of meta-analyses are often used to guide health care policy and to make decisions regarding the management of individual patients. Thus, it is important that the conclusions of meta-analyses are valid. Although the two approaches (meta- and mega-analysis) often produce similar results, sometimes clinical and/or statistical conclusions are affected (Burke et al., 2017). We agree with Burke et al. (2017) and Debray et al. (2013) that when planning IPD analyses in a multi-center setting, the choice and implementation of a mega-analysis (one-stage approach) or meta-analysis (two-stage approach) method should be pre-specified, as occasionally they lead to different conclusions. Standardized statistical guidelines addressing the best approach, such as those mentioned in Burke et al. (2017), would be beneficial in this area. For example, meta-analysis (two-stage approach) or mega-analysis (one-stage approach) may be more suitable, depending on outcome types (continuous, binary of time-to-event). In a multi-center study including multiple small sample cohorts, a mega-analysis (one-stage approach) is preferred, as it avoids the use of approximate normal sampling distributions, known within-study variances, and continuity corrections that plague mega-analysis (two-stage approach) with an inverse variance weighting. Additionally, any mega-analysis (one-stage approach) should account for the clustering of participants within cohorts, ideally by including a random-intercept term for cohort. If the effect sizes of the separate studies are expected to vary greatly, it should be investigated whether adding a random-slope to the model is beneficial. For further details about choosing an appropriate method for a multi-center study we recommend Burke et al. (2017).

To our knowledge, this is the first report investigating the utility of meta- vs. mega-analyses for multi-center structural neuroimaging data. The validity of our findings is limited to cortical gray matter measures. Therefore, they may not be generalized to all other brain measures. Nevertheless, our findings show that in the case of cross-sectional structural neuroimaging data a mega-analysis performs better than a meta-analysis. In a multi-center study with a moderate amount of variation between cohorts, a linear mixed-effects random-intercept mega-analytical framework seems to be the better approach to investigate structural neuroimaging data. We urge researchers worldwide to join forces by sharing data with the goal of elucidating biomedical problems that no group could address alone.

## Ethics Statement

All subjects gave written informed consent in accordance with the Declaration of Helsinki. All local institutional review boards permitted the use of measures extracted from the coded data for meta- and mega-analysis.

## Author Contributions

PB, MH, LS, OvdH, and JT contributed to the conception and design of the study. OvdH and JT contributed equally. PB organized the database. PB and MH performed the statistical analysis at the mega- and mega-analysis level. All other authors contributed to data processing and/or statistical analysis at site level. PB wrote the first draft of the manuscript. All other authors and members of the ENIGMA-OCD working group contributed to manuscript revision, read, and approved the manuscript.

### Conflict of Interest Statement

The authors declare that the research was conducted in the absence of any commercial or financial relationships that could be construed as a potential conflict of interest.
