# Supplementary material for: An Empirical Comparison of Meta- and Mega-Analysis With Data From the ENIGMA Obsessive-Compulsive Disorder Working Group
Source: Front Neuroinform. 2019 Jan 8;12:102. doi: 10.3389/fninf.2018.00102 (PMC6331928; doi:10.3389/fninf.2018.00102)
Supplement: Supplementary file 1 [file Data_Sheet_1.PDF]

Supplementary Tables **Page**
Table of contents

**Supplementary table S1:** Meta-analytic results for cortical thickness of each structure for the adult OCD patients compared with adult healthy controls, controlling for age, sex and scan center.	2
**Supplementary table S2:** Meta-analytic results for cortical surface area of each structure for the adult OCD patients compared with adult healthy controls, controlling for age, sex, scan center and ICV.	4
**Supplementary table S3:** Meta-analytic results for cortical thickness of each structure for the pediatric OCD patients compared with pediatric healthy controls, controlling for age, sex and scan center.	6
**Supplementary table S4:** Meta-analytic results for cortical surface area of each structure for the pediatric OCD patients compared with pediatric healthy controls, controlling for age, sex, scan center and ICV.	8
**Supplementary table S5:** Full mega-analytic results for cortical thickness of each structure for the adult OCD patients compared with adult healthy controls, controlling for age, sex and scan center.	10
**Supplementary table S6:** Full mega-analytic results for cortical surface area of each structure for the adult OCD patients compared with adult healthy controls, controlling for age, sex, scan center, and ICV.	12
**Supplementary table S7:** Full mega-analytic results for cortical thickness of each structure for the pediatric OCD patients compared with pediatric healthy controls, controlling for age, sex and scan center.	14
**Supplementary table S8:** Full mega-analytic results for cortical surface area of each structure for the pediatric OCD patients compared with pediatric healthy controls, controlling for age, sex, scan center, and ICV.	16
**Supplementary table S9:** Comparisons of goodness-of-fit between the different mega-analysis cortical thickness models of the adult sample.	18
**Supplementary table S10:** Comparisons of goodness-of-fit between the different mega-analysis cortical surface area models of the adult sample.	20
**Supplementary table S11:** Comparisons of goodness-of-fit between the different mega-analysis cortical thickness models of the pediatric sample.	22
**Supplementary table S12:** Comparisons of goodness-of-fit between the different mega-analysis cortical surface area models of the pediatric sample.	24

Supplementary table S1: Meta-analytic results for cortical thickness of each structure for the adult OCD patients compared with adult healthy controls, controlling for age, sex and scan center.

	Cohen's d (OCD vs HC)	Standard Error	95% Confidence Interval			P-value
**Left inferior parietal cortex**	-0,133	0,053	-0,237	–	-0,029	0,012
**Left middle temporal gyrus**	-0,132	0,059	-0,247	–	-0,017	0,024
**Right inferior parietal cortex**	-0,132	0,046	-0,221	–	-0,042	0,004
**Right middle temporal gyrus**	-0,109	0,062	-0,231	–	0,013	0,079
**Right medial orbitofrontal cortex**	-0,109	0,045	-0,196	–	-0,022	0,015
**Left rostral middle frontal gyrus**	-0,099	0,047	-0,192	–	-0,007	0,036
**Right fusiform gyrus**	-0,098	0,054	-0,203	–	0,007	0,067
**Right lateral orbitofrontal cortex**	-0,095	0,045	-0,184	–	-0,007	0,035
**Left thickness**	-0,095	0,053	-0,199	–	0,009	0,074
**Left inferior temporal gyrus**	-0,094	0,046	-0,185	–	-0,003	0,042
**Left fusiform gyrus**	-0,094	0,056	-0,203	–	0,015	0,091
**Right rostral middle frontal gyrus**	-0,091	0,048	-0,186	–	0,003	0,058
**Left medial orbitofrontal cortex**	-0,090	0,046	-0,179	–	-0,001	0,048
**Right precuneus cortex**	-0,087	0,057	-0,198	–	0,024	0,125
**Left rostral anterior cingulate cortex**	-0,083	0,038	-0,158	–	-0,009	0,028
**Left precuneus cortex**	-0,079	0,054	-0,185	–	0,027	0,146
**Right parahippocampal gyrus**	-0,078	0,055	-0,186	–	0,029	0,153
**Right thickness**	-0,075	0,056	-0,185	–	0,036	0,185
**Right lateral occipital cortex**	-0,073	0,041	-0,154	–	0,008	0,079
**Left caudal middle frontal gyrus**	-0,072	0,045	-0,160	–	0,015	0,104
**Left lateral orbitofrontal cortex**	-0,072	0,050	-0,170	–	0,025	0,146
**Right caudal middle frontal gyrus**	-0,070	0,049	-0,167	–	0,027	0,155
**Left posterior cingulate cortex**	-0,070	0,044	-0,156	–	0,015	0,108
**Left lateral occipital cortex**	-0,065	0,046	-0,156	–	0,025	0,158
**Left banks superior temporal sulcus**	-0,065	0,043	-0,149	–	0,020	0,133
**Left pars opercularis**	-0,064	0,043	-0,149	–	0,021	0,139
**Left entorhinal cortex**	-0,063	0,053	-0,166	–	0,040	0,230
**Left insula**	-0,057	0,046	-0,148	–	0,033	0,215
**Left superior frontal gyrus**	-0,056	0,056	-0,167	–	0,054	0,317
**Right insula**	-0,055	0,048	-0,150	–	0,039	0,253
**Left isthmus cingulate cortex**	-0,054	0,051	-0,153	–	0,045	0,283
**Right inferior temporal gyrus**	-0,053	0,039	-0,130	–	0,023	0,169
**Left pars orbitalis**	-0,052	0,040	-0,129	–	0,026	0,191
**Right pars triangularis**	-0,050	0,051	-0,150	–	0,051	0,334
**Right posterior cingulate cortex**	-0,050	0,048	-0,143	–	0,044	0,297
**Right pars orbitalis**	-0,046	0,043	-0,131	–	0,039	0,286
**Right isthmus cingulate cortex**	-0,044	0,053	-0,148	–	0,060	0,406
**Left superior parietal cortex**	-0,044	0,061	-0,164	–	0,077	0,476
**Left parahippocampal gyrus**	-0,043	0,046	-0,133	–	0,046	0,340
**Left supramarginal gyrus**	-0,040	0,052	-0,143	–	0,063	0,444

**Right superior parietal cortex**	-0,039	0,055	-0,147	–	0,069	0,480
**Right pars opercularis**	-0,038	0,049	-0,134	–	0,057	0,428
**Left pars triangularis**	-0,036	0,043	-0,119	–	0,047	0,398
**Left frontal pole**	-0,034	0,045	-0,123	–	0,055	0,453
**Right superior frontal gyrus**	-0,029	0,056	-0,138	–	0,081	0,608
**Right transverse temporal cortex**	-0,028	0,038	-0,102	–	0,046	0,460
**Left precentral gyrus**	-0,024	0,046	-0,114	–	0,065	0,592
**Left lingual gyrus**	-0,019	0,047	-0,111	–	0,073	0,685
**Right caudal anterior cingulate cortex**	-0,019	0,045	-0,106	–	0,069	0,675
**Right cuneus cortex**	-0,015	0,044	-0,102	–	0,072	0,735
**Right precentral gyrus**	-0,008	0,057	-0,120	–	0,104	0,885
**Left cuneus cortex**	-0,008	0,049	-0,104	–	0,089	0,879
**Left superior temporal gyrus**	-0,004	0,049	-0,100	–	0,092	0,931
**Right banks superior temporal sulcus**	-0,004	0,052	-0,106	–	0,098	0,939
**Right lingual gyrus**	-0,002	0,048	-0,095	–	0,092	0,972
**Left postcentral gyrus**	-0,002	0,050	-0,100	–	0,097	0,975
**Left caudal anterior cingulate cortex**	-0,001	0,038	-0,075	–	0,074	0,986
**Left paracentral lobule**	0,011	0,054	-0,095	–	0,116	0,845
**Right superior temporal gyrus**	0,013	0,039	-0,064	–	0,090	0,743
**Right frontal pole**	0,013	0,038	-0,061	–	0,087	0,731
**Right paracentral lobule**	0,016	0,042	-0,067	–	0,099	0,700
**Right rostral anterior cingulate cortex**	0,017	0,044	-0,068	–	0,103	0,690
**Left transverse temporal cortex**	0,018	0,046	-0,072	–	0,108	0,692
**Right entorhinal cortex**	0,023	0,051	-0,076	–	0,122	0,650
**Right supramarginal gyrus**	0,027	0,061	-0,092	–	0,146	0,656
**Right temporal pole**	0,027	0,039	-0,049	–	0,104	0,483
**Left temporal pole**	0,029	0,038	-0,045	–	0,103	0,437
**Left pericalcarine cortex**	0,056	0,051	-0,043	–	0,155	0,270
**Right pericalcarine cortex**	0,059	0,048	-0,036	–	0,153	0,223
**Right postcentral gyrus**	0,062	0,063	-0,061	–	0,184	0,326

Supplementary table S2: Full meta-analytic results for cortical surface area of each structure for the adult OCD patients compared with adult healthy controls, controlling for age, sex, scan center and ICV.

	Cohen's d (OCD vs HC)	Standard Error	95% Confidence Interval			P-value
**Left transverse temporal cortex**	-0,169	0,038	-0,243	–	-0,095	7,623E-06
**Right superior temporal gyrus**	-0,125	0,039	-0,202	–	-0,047	0,002
**Right transverse temporal cortex**	-0,107	0,038	-0,181	–	-0,033	0,005
**Right supramarginal gyrus**	-0,100	0,048	-0,194	–	-0,007	0,036
**Left pars opercularis**	-0,099	0,038	-0,174	–	-0,025	0,009
**Left pars triangularis**	-0,098	0,038	-0,172	–	-0,024	0,009
**Right posterior cingulate cortex**	-0,090	0,064	-0,215	–	0,035	0,159
**Left superior temporal gyrus**	-0,084	0,040	-0,162	–	-0,005	0,036
**Left superior parietal cortex**	-0,081	0,050	-0,178	–	0,017	0,104
**Left superior frontal gyrus**	-0,080	0,038	-0,155	–	-0,004	0,038
**Left surface area**	-0,078	0,038	-0,152	–	-0,004	0,039
**Right rostral anterior cingulate cortex**	-0,075	0,046	-0,165	–	0,015	0,104
**Right superior frontal gyrus**	-0,072	0,040	-0,151	–	0,007	0,072
**Left postcentral gyrus**	-0,070	0,038	-0,145	–	0,005	0,067
**Left supramarginal gyrus**	-0,068	0,040	-0,145	–	0,010	0,087
**Left lateral orbitofrontal cortex**	-0,059	0,048	-0,153	–	0,035	0,220
**Right superior parietal cortex**	-0,056	0,049	-0,152	–	0,041	0,258
**Left lateral occipital cortex**	-0,055	0,052	-0,156	–	0,047	0,289
**Right surface area**	-0,053	0,038	-0,127	–	0,021	0,158
**Left insula**	-0,050	0,047	-0,143	–	0,043	0,291
**Left frontal pole**	-0,048	0,053	-0,151	–	0,056	0,365
**Right caudal anterior cingulate cortex**	-0,048	0,056	-0,157	–	0,062	0,392
**Right pars opercularis**	-0,046	0,038	-0,120	–	0,029	0,230
**Right fusiform gyrus**	-0,042	0,055	-0,150	–	0,066	0,443
**Right middle temporal gyrus**	-0,042	0,038	-0,117	–	0,032	0,268
**Left posterior cingulate cortex**	-0,040	0,053	-0,143	–	0,063	0,446
**Left inferior temporal gyrus**	-0,039	0,038	-0,114	–	0,036	0,310
**Right lateral occipital cortex**	-0,039	0,038	-0,113	–	0,036	0,309
**Right pericalcarine cortex**	-0,038	0,038	-0,113	–	0,036	0,312
**Left caudal middle frontal gyrus**	-0,036	0,044	-0,123	–	0,050	0,408
**Right isthmus cingulate cortex**	-0,031	0,040	-0,109	–	0,047	0,432
**Left isthmus cingulate cortex**	-0,029	0,047	-0,121	–	0,063	0,536
**Left pars orbitalis**	-0,028	0,038	-0,102	–	0,046	0,453
**Right lingual gyrus**	-0,025	0,038	-0,099	–	0,049	0,512
**Right insula**	-0,024	0,044	-0,110	–	0,062	0,582
**Left lingual gyrus**	-0,022	0,048	-0,117	–	0,073	0,645
**Left paracentral lobule**	-0,021	0,044	-0,108	–	0,066	0,633
**Left entorhinal cortex**	-0,020	0,051	-0,120	–	0,081	0,700
**Right pars triangularis**	-0,018	0,047	-0,111	–	0,074	0,697
**Left pericalcarine cortex**	-0,017	0,038	-0,091	–	0,058	0,664

**Left rostral anterior cingulate cortex**	-0,013	0,053	-0,116	–	0,091	0,810
**Right precentral gyrus**	-0,013	0,038	-0,087	–	0,062	0,742
**Left medial orbitofrontal cortex**	-0,011	0,038	-0,085	–	0,063	0,766
**Left cuneus cortex**	-0,009	0,041	-0,090	–	0,072	0,829
**Right postcentral gyrus**	-0,008	0,043	-0,093	–	0,077	0,856
**Left temporal pole**	-0,005	0,038	-0,079	–	0,069	0,902
**Right cuneus cortex**	-0,004	0,052	-0,107	–	0,098	0,936
**Left precentral gyrus**	-0,002	0,038	-0,076	–	0,073	0,962
**Left middle temporal gyrus**	-0,002	0,039	-0,079	–	0,076	0,968
**Right pars orbitalis**	-0,001	0,051	-0,101	–	0,099	0,982
**Right caudal middle frontal gyrus**	0,000	0,042	-0,083	–	0,082	0,995
**Right precuneus cortex**	0,005	0,057	-0,107	–	0,116	0,936
**Right paracentral lobule**	0,008	0,038	-0,066	–	0,082	0,840
**Right rostral middle frontal gyrus**	0,010	0,044	-0,075	–	0,095	0,819
**Left rostral middle frontal gyrus**	0,011	0,045	-0,077	–	0,100	0,802
**Right lateral orbitofrontal cortex**	0,011	0,038	-0,063	–	0,085	0,762
**Left fusiform gyrus**	0,013	0,038	-0,062	–	0,087	0,740
**Right parahippocampal gyrus**	0,013	0,052	-0,088	–	0,114	0,803
**Left banks superior temporal sulcus**	0,014	0,040	-0,064	–	0,091	0,733
**Left caudal anterior cingulate cortex**	0,014	0,042	-0,068	–	0,097	0,733
**Right temporal pole**	0,016	0,052	-0,086	–	0,118	0,759
**Right frontal pole**	0,017	0,038	-0,057	–	0,090	0,661
**Right inferior temporal gyrus**	0,024	0,042	-0,059	–	0,106	0,576
**Right banks superior temporal sulcus**	0,024	0,039	-0,052	–	0,101	0,535
**Right medial orbitofrontal cortex**	0,024	0,038	-0,050	–	0,099	0,518
**Left inferior parietal cortex**	0,028	0,051	-0,072	–	0,128	0,581
**Right entorhinal cortex**	0,030	0,057	-0,083	–	0,142	0,605
**Left parahippocampal gyrus**	0,034	0,038	-0,040	–	0,108	0,374
**Left precuneus cortex**	0,040	0,044	-0,045	–	0,126	0,358
**Right inferior parietal cortex**	0,073	0,049	-0,023	–	0,170	0,138

Supplementary table S3: Meta-analytic results for cortical thickness of each structure for the pediatric OCD patients compared with pediatric healthy controls, controlling for age, sex and scan center.

	Cohen's d (OCD vs HC)	Standard Error	95% Confidence Interval			P-value
**Left inferior parietal cortex**	-0,310	0,144	-0,593	–	-0,027	0,032
**Left superior parietal cortex**	-0,281	0,159	-0,593	–	0,030	0,076
**Right superior parietal cortex**	-0,271	0,138	-0,540	–	-0,001	0,049
**Left lateral occipital cortex**	-0,258	0,095	-0,445	–	-0,071	0,007
**Left cuneus cortex**	-0,223	0,092	-0,402	–	-0,043	0,015
**Left precuneus cortex**	-0,179	0,142	-0,458	–	0,100	0,208
**Left thickness**	-0,175	0,127	-0,423	–	0,073	0,167
**Left pericalcarine cortex**	-0,173	0,077	-0,324	–	-0,022	0,024
**Right inferior parietal cortex**	-0,173	0,173	-0,513	–	0,167	0,319
**Right lateral occipital cortex**	-0,163	0,123	-0,404	–	0,077	0,183
**Left banks superior temporal sulcus**	-0,163	0,086	-0,332	–	0,006	0,058
**Left posterior cingulate cortex**	-0,160	0,114	-0,384	–	0,064	0,162
**Left supramarginal gyrus**	-0,155	0,118	-0,386	–	0,076	0,189
**Right precuneus cortex**	-0,154	0,117	-0,383	–	0,075	0,188
**Left medial orbitofrontal cortex**	-0,149	0,077	-0,301	–	0,002	0,053
**Left paracentral lobule**	-0,148	0,114	-0,371	–	0,075	0,194
**Right cuneus cortex**	-0,143	0,087	-0,314	–	0,029	0,102
**Right thickness**	-0,138	0,120	-0,372	–	0,096	0,248
**Left isthmus cingulate cortex**	-0,128	0,101	-0,326	–	0,069	0,202
**Right caudal middle frontal gyrus**	-0,128	0,110	-0,342	–	0,087	0,244
**Right pericalcarine cortex**	-0,112	0,083	-0,276	–	0,052	0,180
**Right inferior temporal gyrus**	-0,110	0,077	-0,261	–	0,042	0,156
**Left inferior temporal gyrus**	-0,106	0,097	-0,295	–	0,084	0,275
**Right posterior cingulate cortex**	-0,102	0,120	-0,337	–	0,133	0,397
**Left pars opercularis**	-0,099	0,111	-0,316	–	0,118	0,372
**Right insula**	-0,097	0,078	-0,250	–	0,056	0,212
**Right postcentral gyrus**	-0,097	0,078	-0,249	–	0,055	0,210
**Right paracentral lobule**	-0,097	0,092	-0,278	–	0,084	0,296
**Left postcentral gyrus**	-0,096	0,103	-0,298	–	0,106	0,352
**Right banks superior temporal sulcus**	-0,095	0,096	-0,284	–	0,093	0,322
**Left fusiform gyrus**	-0,095	0,121	-0,333	–	0,142	0,432
**Left precentral gyrus**	-0,091	0,113	-0,312	–	0,130	0,418
**Left transverse temporal cortex**	-0,088	0,113	-0,310	–	0,134	0,436
**Right rostral anterior cingulate cortex**	-0,087	0,078	-0,240	–	0,067	0,268
**Left caudal middle frontal gyrus**	-0,084	0,133	-0,344	–	0,176	0,527
**Right middle temporal gyrus**	-0,081	0,120	-0,315	–	0,154	0,499
**Right pars triangularis**	-0,080	0,087	-0,250	–	0,089	0,353
**Left lingual gyrus**	-0,075	0,077	-0,226	–	0,076	0,329
**Left middle temporal gyrus**	-0,073	0,136	-0,339	–	0,193	0,589
**Right rostral middle frontal gyrus**	-0,073	0,077	-0,223	–	0,078	0,344

**Left frontal pole**	-0,065	0,077	-0,215	–	0,086	0,399
**Right pars opercularis**	-0,063	0,077	-0,214	–	0,088	0,414
**Right transverse temporal cortex**	-0,062	0,086	-0,230	–	0,106	0,468
**Right pars orbitalis**	-0,058	0,077	-0,209	–	0,093	0,450
**Left superior frontal gyrus**	-0,056	0,077	-0,207	–	0,096	0,473
**Right precentral gyrus**	-0,054	0,112	-0,274	–	0,165	0,627
**Right parahippocampal gyrus**	-0,050	0,078	-0,203	–	0,102	0,517
**Right supramarginal gyrus**	-0,048	0,119	-0,280	–	0,185	0,688
**Left pars orbitalis**	-0,046	0,077	-0,197	–	0,105	0,548
**Left rostral middle frontal gyrus**	-0,043	0,077	-0,193	–	0,108	0,577
**Left pars triangularis**	-0,038	0,077	-0,188	–	0,113	0,623
**Right isthmus cingulate cortex**	-0,036	0,077	-0,188	–	0,115	0,637
**Right temporal pole**	-0,033	0,087	-0,203	–	0,137	0,704
**Right superior frontal gyrus**	-0,032	0,081	-0,190	–	0,126	0,690
**Right fusiform gyrus**	-0,024	0,109	-0,238	–	0,190	0,827
**Left parahippocampal gyrus**	-0,021	0,092	-0,201	–	0,159	0,819
**Right superior temporal gyrus**	-0,006	0,114	-0,230	–	0,217	0,957
**Right lateral orbitofrontal cortex**	-0,004	0,077	-0,155	–	0,146	0,954
**Left superior temporal gyrus**	-0,004	0,089	-0,180	–	0,171	0,961
**Left insula**	-0,004	0,099	-0,199	–	0,190	0,965
**Left temporal pole**	-0,003	0,078	-0,157	–	0,150	0,968
**Right entorhinal cortex**	0,000	0,106	-0,208	–	0,207	0,998
**Right caudal anterior cingulate cortex**	0,012	0,101	-0,186	–	0,209	0,909
**Right lingual gyrus**	0,015	0,077	-0,135	–	0,166	0,843
**Left caudal anterior cingulate cortex**	0,016	0,082	-0,145	–	0,177	0,846
**Left entorhinal cortex**	0,031	0,117	-0,198	–	0,260	0,789
**Right frontal pole**	0,038	0,077	-0,113	–	0,189	0,626
**Left lateral orbitofrontal cortex**	0,063	0,077	-0,087	–	0,213	0,412
**Left rostral anterior cingulate cortex**	0,081	0,078	-0,071	–	0,233	0,298
**Right medial orbitofrontal cortex**	0,094	0,077	-0,057	–	0,246	0,222

Supplementary table S4: Full meta-analytic results for cortical surface area of each structure for the pediatric OCD patients compared with pediatric healthy controls, controlling for age, sex, scan center and ICV.

	Cohen's d (OCD vs HC)	Standard Error	95% Confidence Interval			P-value
**Right paracentral lobule**	-0,219	0,076	-0,368	–	-0,071	0,004
**Left posterior cingulate cortex**	-0,203	0,079	-0,359	–	-0,048	0,010
**Right medial orbitofrontal cortex**	-0,200	0,141	-0,477	–	0,077	0,158
**Right superior frontal gyrus**	-0,181	0,113	-0,402	–	0,041	0,109
**Left lateral orbitofrontal cortex**	-0,171	0,076	-0,320	–	-0,023	0,024
**Left rostral middle frontal gyrus**	-0,154	0,114	-0,379	–	0,070	0,177
**Left medial orbitofrontal cortex**	-0,145	0,076	-0,295	–	0,004	0,057
**Left postcentral gyrus**	-0,134	0,077	-0,285	–	0,017	0,082
**Right fusiform gyrus**	-0,124	0,092	-0,304	–	0,056	0,178
**Left paracentral lobule**	-0,123	0,121	-0,360	–	0,114	0,307
**Left superior frontal gyrus**	-0,112	0,110	-0,327	–	0,103	0,307
**Right pars triangularis**	-0,110	0,082	-0,270	–	0,051	0,181
**Right rostral middle frontal gyrus**	-0,108	0,092	-0,289	–	0,073	0,243
**Right pars orbitalis**	-0,106	0,076	-0,255	–	0,042	0,160
**Left pericalcarine cortex**	-0,104	0,086	-0,272	–	0,064	0,226
**Left inferior parietal cortex**	-0,103	0,116	-0,330	–	0,124	0,374
**Right cuneus cortex**	-0,097	0,076	-0,246	–	0,051	0,200
**Right lateral orbitofrontal cortex**	-0,095	0,080	-0,252	–	0,061	0,233
**Right posterior cingulate cortex**	-0,092	0,095	-0,279	–	0,095	0,335
**Right superior parietal cortex**	-0,092	0,111	-0,309	–	0,125	0,408
**Left caudal anterior cingulate cortex**	-0,088	0,083	-0,251	–	0,075	0,292
**Left pars orbitalis**	-0,085	0,077	-0,235	–	0,066	0,270
**Right lateral occipital cortex**	-0,085	0,082	-0,246	–	0,076	0,303
**Right surface area**	-0,083	0,101	-0,281	–	0,114	0,407
**Left cuneus cortex**	-0,081	0,076	-0,230	–	0,068	0,286
**Right inferior parietal cortex**	-0,073	0,077	-0,224	–	0,077	0,340
**Left insula**	-0,066	0,082	-0,227	–	0,096	0,426
**Left precuneus cortex**	-0,055	0,104	-0,260	–	0,150	0,598
**Left frontal pole**	-0,049	0,076	-0,198	–	0,099	0,513
**Left superior parietal cortex**	-0,046	0,089	-0,220	–	0,129	0,610
**Left pars opercularis**	-0,045	0,118	-0,278	–	0,187	0,701
**Right pericalcarine cortex**	-0,044	0,076	-0,193	–	0,104	0,559
**Right lingual gyrus**	-0,042	0,076	-0,190	–	0,106	0,579
**Right insula**	-0,036	0,077	-0,187	–	0,116	0,645
**Left surface area**	-0,035	0,125	-0,281	–	0,210	0,778
**Right postcentral gyrus**	-0,028	0,077	-0,179	–	0,123	0,715
**Left banks superior temporal sulcus**	-0,024	0,085	-0,190	–	0,143	0,780
**Right parahippocampal gyrus**	-0,023	0,076	-0,173	–	0,126	0,758
**Right superior temporal gyrus**	-0,023	0,109	-0,238	–	0,191	0,831

**Left middle temporal gyrus**	-0,023	0,193	-0,402	–	0,356	0,905
**Left rostral anterior cingulate cortex**	-0,021	0,130	-0,275	–	0,233	0,871
**Right middle temporal gyrus**	-0,017	0,101	-0,215	–	0,182	0,869
**Right supramarginal gyrus**	-0,016	0,078	-0,168	–	0,137	0,840
**Left fusiform gyrus**	-0,012	0,093	-0,194	–	0,170	0,896
**Left lingual gyrus**	-0,010	0,076	-0,159	–	0,138	0,891
**Right temporal pole**	-0,001	0,076	-0,149	–	0,148	0,994
**Right pars opercularis**	0,005	0,076	-0,144	–	0,155	0,945
**Left entorhinal cortex**	0,010	0,080	-0,146	–	0,166	0,901
**Right rostral anterior cingulate cortex**	0,012	0,077	-0,138	–	0,163	0,872
**Right caudal middle frontal gyrus**	0,014	0,118	-0,216	–	0,245	0,904
**Right precuneus cortex**	0,019	0,102	-0,182	–	0,219	0,855
**Left caudal middle frontal gyrus**	0,024	0,160	-0,289	–	0,337	0,882
**Left parahippocampal gyrus**	0,025	0,120	-0,209	–	0,260	0,833
**Left superior temporal gyrus**	0,026	0,094	-0,157	–	0,210	0,778
**Right banks superior temporal sulcus**	0,026	0,079	-0,129	–	0,181	0,738
**Right caudal anterior cingulate cortex**	0,030	0,091	-0,147	–	0,208	0,738
**Left lateral occipital cortex**	0,034	0,082	-0,127	–	0,196	0,676
**Left supramarginal gyrus**	0,042	0,098	-0,151	–	0,235	0,670
**Left transverse temporal cortex**	0,042	0,087	-0,128	–	0,213	0,625
**Left pars triangularis**	0,051	0,076	-0,098	–	0,199	0,504
**Right inferior temporal gyrus**	0,053	0,134	-0,211	–	0,316	0,695
**Right precentral gyrus**	0,053	0,113	-0,168	–	0,274	0,639
**Left precentral gyrus**	0,062	0,085	-0,104	–	0,229	0,462
**Right isthmus cingulate cortex**	0,074	0,099	-0,121	–	0,268	0,458
**Right transverse temporal cortex**	0,089	0,109	-0,125	–	0,304	0,414
**Left inferior temporal gyrus**	0,091	0,171	-0,244	–	0,426	0,596
**Left temporal pole**	0,111	0,076	-0,038	–	0,260	0,144
**Right frontal pole**	0,131	0,109	-0,081	–	0,344	0,226
**Right entorhinal cortex**	0,148	0,130	-0,107	–	0,403	0,256
**Left isthmus cingulate cortex**	0,149	0,076	0,000	–	0,298	0,051

Supplementary table S5: Full mega-analytic results for cortical thickness of each structure for the adult OCD patients compared with adult healthy controls, controlling for age, sex and scan center.

	Cohen's d		P-value		Standard Error		95% Confidence Interval					
	LR	LMEri	LR	LMEri	LR	LMEri	LR			LMEri		
**Left inferior parietal cortex**	**-0,14**	**-0,11**	**<0,001**	**<0,001**	**0,038**	**0,038**	**-0,211**	**–**	**-0,063**	**-0,214**	**–**	**-0,065**
**Right inferior parietal cortex**	**-0,14**	**-0,10**	**<0,001**	**<0,001**	**0,038**	**0,038**	**-0,211**	**–**	**-0,064**	**-0,213**	**–**	**-0,066**
**Right medial orbitofrontal cortex**	-0,10	-0,09	0,006	0,006	0,037	0,037	-0,176	–	-0,030	-0,175	–	-0,030
**Left lateral orbitofrontal cortex**	-0,10	-0,08	0,008	0,008	0,037	0,037	-0,172	–	-0,027	-0,171	–	-0,026
**Right lateral orbitofrontal cortex**	-0,11	-0,08	0,003	0,003	0,037	0,037	-0,181	–	-0,036	-0,181	–	-0,036
**Left precuneus cortex**	-0,10	-0,08	0,008	0,008	0,037	0,037	-0,170	–	-0,025	-0,171	–	-0,026
**Left fusiform gyrus**	-0,11	-0,08	0,003	0,004	0,037	0,037	-0,181	–	-0,036	-0,181	–	-0,036
**Left thickness**	-0,11	-0,08	0,004	0,004	0,037	0,037	-0,178	–	-0,033	-0,180	–	-0,035
**Right precuneus cortex**	-0,10	-0,07	0,006	0,006	0,037	0,037	-0,172	–	-0,027	-0,174	–	-0,029
**Right middle temporal gyrus**	-0,10	-0,07	0,009	0,010	0,037	0,037	-0,171	–	-0,024	-0,170	–	-0,024
**Left middle temporal gyrus**	-0,09	-0,07	0,016	0,019	0,039	0,039	-0,168	–	-0,016	-0,167	–	-0,015
**Left medial orbitofrontal cortex**	-0,08	-0,07	0,023	0,023	0,037	0,037	-0,157	–	-0,012	-0,157	–	-0,012
**Right rostral middle frontal gyrus**	-0,09	-0,07	0,014	0,014	0,037	0,037	-0,162	–	-0,017	-0,164	–	-0,019
**Left rostral middle frontal gyrus**	-0,10	-0,07	0,010	0,010	0,037	0,037	-0,167	–	-0,022	-0,168	–	-0,023
**Left inferior temporal gyrus**	-0,09	-0,07	0,020	0,022	0,037	0,037	-0,160	–	-0,013	-0,160	–	-0,013
**Left pars opercularis**	-0,08	-0,07	0,025	0,025	0,037	0,037	-0,155	–	-0,010	-0,156	–	-0,011
**Right thickness**	-0,09	-0,07	0,012	0,012	0,037	0,037	-0,164	–	-0,019	-0,166	–	-0,021
**Left caudal middle frontal gyrus**	-0,09	-0,07	0,015	0,015	0,037	0,037	-0,161	–	-0,016	-0,163	–	-0,018
**Left rostral anterior cingulate cortex**	-0,08	-0,07	0,044	0,047	0,037	0,037	-0,148	–	-0,002	-0,147	–	-0,001
**Right parahippocampal gyrus**	-0,08	-0,07	0,029	0,033	0,037	0,037	-0,155	–	-0,010	-0,152	–	-0,007
**Right caudal middle frontal gyrus**	-0,08	-0,06	0,031	0,031	0,037	0,037	-0,152	–	-0,006	-0,153	–	-0,008
**Right fusiform gyrus**	-0,09	-0,06	0,015	0,015	0,037	0,037	-0,164	–	-0,018	-0,164	–	-0,018
**Left isthmus cingulate cortex**	-0,07	-0,06	0,064	0,068	0,037	0,037	-0,142	–	0,003	-0,140	–	0,005
**Left posterior cingulate cortex**	-0,07	-0,06	0,054	0,056	0,037	0,037	-0,143	–	0,002	-0,144	–	0,001
**Right pars orbitalis**	-0,07	-0,06	0,062	0,062	0,037	0,037	-0,141	–	0,004	-0,142	–	0,003
**Left lateral occipital cortex**	-0,07	-0,06	0,045	0,044	0,037	0,037	-0,146	–	0,000	-0,148	–	-0,002
**Left entorhinal cortex**	-0,06	-0,05	0,110	0,116	0,039	0,039	-0,137	–	0,014	-0,136	–	0,015
**Left insula**	-0,07	-0,05	0,076	0,082	0,037	0,037	-0,139	–	0,007	-0,138	–	0,008
**Right lateral occipital cortex**	-0,07	-0,05	0,053	0,052	0,037	0,037	-0,143	–	0,003	-0,145	–	0,000
**Right insula**	-0,07	-0,05	0,077	0,081	0,037	0,037	-0,139	–	0,007	-0,138	–	0,008
**Left parahippocampal gyrus**	-0,06	-0,05	0,104	0,113	0,037	0,037	-0,135	–	0,011	-0,132	–	0,014
**Right pars opercularis**	-0,06	-0,05	0,092	0,095	0,037	0,037	-0,135	–	0,011	-0,135	–	0,010
**Right pars triangularis**	-0,06	-0,05	0,126	0,126	0,037	0,037	-0,129	–	0,017	-0,130	–	0,016
**Left superior frontal gyrus**	-0,07	-0,05	0,067	0,067	0,037	0,037	-0,140	–	0,007	-0,142	–	0,004
**Left banks superior temporal sulcus**	-0,06	-0,05	0,125	0,136	0,039	0,039	-0,136	–	0,017	-0,135	–	0,018
**Left superior parietal cortex**	-0,06	-0,05	0,096	0,094	0,037	0,037	-0,132	–	0,014	-0,136	–	0,010
**Right posterior cingulate cortex**	-0,06	-0,04	0,133	0,137	0,037	0,037	-0,128	–	0,017	-0,128	–	0,017
**Left pars orbitalis**	-0,05	-0,04	0,177	0,177	0,037	0,037	-0,123	–	0,022	-0,123	–	0,022
**Right inferior temporal gyrus**	-0,06	-0,04	0,111	0,119	0,037	0,037	-0,132	–	0,014	-0,131	–	0,015
**Right isthmus cingulate cortex**	-0,05	-0,04	0,154	0,162	0,037	0,037	-0,126	–	0,019	-0,125	–	0,020
**Left lingual gyrus**	-0,05	-0,04	0,152	0,147	0,037	0,037	-0,127	–	0,018	-0,127	–	0,019
**Left frontal pole**	-0,05	-0,04	0,191	0,192	0,037	0,037	-0,120	–	0,025	-0,121	–	0,024
**Left supramarginal gyrus**	-0,05	-0,04	0,179	0,184	0,039	0,039	-0,126	–	0,025	-0,128	–	0,024
**Left cuneus cortex**	-0,04	-0,03	0,261	0,252	0,037	0,037	-0,115	–	0,031	-0,116	–	0,030

**Right superior parietal cortex**	-0,05	-0,03	0,207	0,202	0,037	0,037	-0,117	–	0,029	-0,121	–	0,025
**Right lingual gyrus**	-0,05	-0,03	0,222	0,217	0,037	0,037	-0,119	–	0,026	-0,118	–	0,027
**Right caudal anterior cingulate cortex**	-0,04	-0,03	0,307	0,321	0,037	0,037	-0,111	–	0,035	-0,110	–	0,036
**Right superior frontal gyrus**	-0,04	-0,03	0,300	0,294	0,037	0,037	-0,111	–	0,035	-0,112	–	0,034
**Left pars triangularis**	-0,03	-0,03	0,369	0,374	0,037	0,037	-0,105	–	0,040	-0,106	–	0,039
**Right cuneus cortex**	-0,03	-0,03	0,362	0,351	0,037	0,037	-0,107	–	0,039	-0,108	–	0,038
**Right precentral gyrus**	-0,04	-0,02	0,318	0,318	0,037	0,037	-0,109	–	0,038	-0,111	–	0,036
**Right transverse temporal cortex**	-0,02	-0,02	0,502	0,511	0,037	0,037	-0,098	–	0,047	-0,097	–	0,048
**Left postcentral gyrus**	-0,02	-0,02	0,538	0,533	0,037	0,037	-0,094	–	0,052	-0,097	–	0,050
**Left precentral gyrus**	-0,02	-0,01	0,507	0,508	0,037	0,037	-0,096	–	0,050	-0,098	–	0,048
**Left paracentral lobule**	-0,01	-0,01	0,727	0,719	0,037	0,037	-0,084	–	0,061	-0,086	–	0,059
**Left superior temporal gyrus**	-0,01	0,00	0,845	0,879	0,039	0,039	-0,084	–	0,069	-0,083	–	0,070
**Left transverse temporal cortex**	0,00	0,00	0,898	0,913	0,037	0,037	-0,078	–	0,067	-0,077	–	0,068
**Right supramarginal gyrus**	0,00	0,00	0,946	0,930	0,038	0,038	-0,071	–	0,080	-0,072	–	0,079
**Right banks superior temporal sulcus**	0,00	0,00	0,944	0,909	0,038	0,038	-0,072	–	0,079	-0,071	–	0,080
**Left caudal anterior cingulate cortex**	0,00	0,00	0,943	0,914	0,037	0,037	-0,070	–	0,076	-0,069	–	0,077
**Right rostral anterior cingulate cortex**	0,00	0,00	0,905	0,906	0,037	0,037	-0,069	–	0,077	-0,069	–	0,077
**Right paracentral lobule**	0,01	0,00	0,843	0,848	0,037	0,037	-0,063	–	0,082	-0,065	–	0,080
**Left pericalcarine cortex**	0,01	0,01	0,752	0,782	0,037	0,037	-0,061	–	0,085	-0,063	–	0,083
**Right superior temporal gyrus**	0,01	0,01	0,760	0,730	0,039	0,039	-0,063	–	0,088	-0,062	–	0,089
**Right entorhinal cortex**	0,01	0,01	0,729	0,720	0,039	0,039	-0,063	–	0,092	-0,063	–	0,091
**Right temporal pole**	0,02	0,01	0,655	0,634	0,037	0,037	-0,056	–	0,090	-0,055	–	0,091
**Right frontal pole**	0,02	0,02	0,597	0,602	0,037	0,037	-0,052	–	0,093	-0,053	–	0,092
**Right pericalcarine cortex**	0,02	0,02	0,516	0,549	0,037	0,037	-0,049	–	0,097	-0,051	–	0,095
**Right postcentral gyrus**	0,04	0,03	0,344	0,348	0,037	0,037	-0,035	–	0,111	-0,038	–	0,108
**Left temporal pole**	0,03	0,03	0,366	0,330	0,037	0,037	-0,039	–	0,106	-0,036	–	0,109

Abbreviations: LR = linear regression; LMEri= linear mixed-effects random-intercept model

Supplementary table S6: Full mega-analytic results for cortical surface area of each structure for the adult OCD patients compared with adult healthy controls, controlling for age, sex, scan center, and ICV.

	Cohen's d		P-value		Standard Error		95% Confidence Interval					
	LR	LMEri	LR	LMEri	LR	LMEri	LR			LMEri		
**Left transverse temporal cortex**	**-0,16**	**-0,16**	**<0,001**	**<0,001**	**0,037**	**0,037**	**-0,238**	**–**	**-0,092**	**-0,240**	**–**	**-0,095**
**Right superior temporal gyrus**	-0,11	-0,11	0,004	0,003	0,039	0,039	-0,188	–	-0,037	-0,189	–	-0,038
**Right transverse temporal cortex**	-0,10	-0,10	0,005	0,004	0,037	0,037	-0,178	–	-0,033	-0,178	–	-0,033
**Left pars triangularis**	-0,10	-0,10	0,010	0,008	0,037	0,037	-0,169	–	-0,024	-0,171	–	-0,026
**Left pars opercularis**	-0,09	-0,09	0,020	0,016	0,037	0,037	-0,160	–	-0,015	-0,162	–	-0,017
**Right supramarginal gyrus**	-0,08	-0,08	0,039	0,035	0,038	0,038	-0,156	–	-0,005	-0,157	–	-0,006
**Left superior temporal gyrus**	-0,07	-0,07	0,066	0,061	0,039	0,039	-0,150	–	0,004	-0,150	–	0,003
**Left postcentral gyrus**	-0,07	-0,07	0,054	0,045	0,037	0,037	-0,146	–	0,001	-0,149	–	-0,002
**Right posterior cingulate cortex**	-0,07	-0,07	0,063	0,054	0,037	0,037	-0,142	–	0,003	-0,144	–	0,001
**Right superior frontal gyrus**	-0,06	-0,06	0,093	0,083	0,037	0,037	-0,138	–	0,009	-0,138	–	0,008
**Right rostral anterior cingulate cortex**	-0,06	-0,06	0,101	0,090	0,037	0,037	-0,135	–	0,011	-0,136	–	0,010
**Left surface area**	-0,06	-0,05	0,108	0,099	0,037	0,037	-0,133	–	0,012	-0,134	–	0,011
**Left superior frontal gyrus**	-0,06	-0,05	0,115	0,104	0,037	0,037	-0,134	–	0,013	-0,135	–	0,012
**Left supramarginal gyrus**	-0,06	-0,05	0,144	0,125	0,039	0,039	-0,133	–	0,019	-0,135	–	0,016
**Left superior parietal cortex**	-0,05	-0,05	0,182	0,159	0,037	0,037	-0,123	–	0,023	-0,126	–	0,020
**Left insula**	-0,05	-0,04	0,188	0,177	0,037	0,037	-0,122	–	0,024	-0,124	–	0,023
**Left lateral orbitofrontal cortex**	-0,05	-0,04	0,186	0,167	0,037	0,037	-0,123	–	0,022	-0,124	–	0,021
**Right caudal anterior cingulate cortex**	-0,05	-0,05	0,199	0,157	0,037	0,037	-0,122	–	0,024	-0,125	–	0,020
**Left lateral occipital cortex**	-0,05	-0,04	0,222	0,204	0,037	0,037	-0,119	–	0,027	-0,120	–	0,026
**Left caudal middle frontal gyrus**	-0,04	-0,04	0,270	0,227	0,037	0,037	-0,115	–	0,030	-0,118	–	0,028
**Right pars opercularis**	-0,04	-0,04	0,272	0,229	0,037	0,037	-0,115	–	0,031	-0,118	–	0,028
**Right surface area**	-0,04	-0,03	0,304	0,285	0,037	0,037	-0,112	–	0,033	-0,112	–	0,033
**Right pericalcarine cortex**	-0,04	-0,03	0,342	0,349	0,037	0,037	-0,108	–	0,039	-0,108	–	0,038
**Left frontal pole**	-0,03	-0,04	0,356	0,310	0,037	0,037	-0,108	–	0,037	-0,110	–	0,035
**Right superior parietal cortex**	-0,03	-0,04	0,360	0,319	0,037	0,037	-0,108	–	0,038	-0,110	–	0,036
**Right middle temporal gyrus**	-0,03	-0,03	0,365	0,346	0,037	0,037	-0,108	–	0,039	-0,109	–	0,038
**Right pars triangularis**	-0,03	-0,03	0,390	0,347	0,037	0,037	-0,106	–	0,040	-0,108	–	0,038
**Left pars orbitalis**	-0,03	-0,03	0,387	0,355	0,037	0,037	-0,106	–	0,039	-0,107	–	0,038
**Right isthmus cingulate cortex**	-0,03	-0,03	0,397	0,358	0,037	0,037	-0,104	–	0,041	-0,107	–	0,038
**Left lingual gyrus**	-0,03	-0,02	0,453	0,454	0,037	0,037	-0,100	–	0,045	-0,100	–	0,045
**Left posterior cingulate cortex**	-0,03	-0,03	0,478	0,438	0,037	0,037	-0,099	–	0,046	-0,101	–	0,044
**Right lateral occipital cortex**	-0,03	-0,03	0,495	0,464	0,037	0,037	-0,099	–	0,047	-0,100	–	0,046
**Left cuneus cortex**	-0,03	-0,02	0,500	0,506	0,037	0,037	-0,098	–	0,048	-0,098	–	0,048
**Left inferior temporal gyrus**	-0,02	-0,02	0,513	0,473	0,037	0,037	-0,099	–	0,048	-0,100	–	0,046
**Right insula**	-0,02	-0,02	0,523	0,509	0,037	0,037	-0,096	–	0,050	-0,098	–	0,048
**Right entorhinal cortex**	-0,02	-0,02	0,576	0,543	0,039	0,039	-0,100	–	0,055	-0,101	–	0,053
**Left isthmus cingulate cortex**	-0,02	-0,02	0,571	0,507	0,037	0,037	-0,094	–	0,051	-0,097	–	0,048
**Right cuneus cortex**	-0,02	-0,02	0,633	0,629	0,037	0,037	-0,091	–	0,055	-0,091	–	0,055
**Left paracentral lobule**	-0,02	-0,02	0,648	0,601	0,037	0,037	-0,091	–	0,054	-0,092	–	0,053
**Left pericalcarine cortex**	-0,02	-0,01	0,680	0,684	0,037	0,037	-0,087	–	0,059	-0,088	–	0,058
**Right postcentral gyrus**	-0,01	-0,02	0,698	0,646	0,037	0,037	-0,088	–	0,058	-0,090	–	0,056
**Left entorhinal cortex**	-0,01	-0,02	0,742	0,681	0,039	0,039	-0,088	–	0,063	-0,091	–	0,060
**Right lingual gyrus**	-0,01	-0,01	0,743	0,754	0,037	0,037	-0,084	–	0,061	-0,084	–	0,061
**Right precuneus cortex**	-0,01	-0,01	0,790	0,744	0,037	0,037	-0,083	–	0,062	-0,085	–	0,060
**Right precentral gyrus**	-0,01	-0,01	0,802	0,736	0,037	0,037	-0,083	–	0,063	-0,086	–	0,061
**Right caudal middle frontal gyrus**	-0,01	-0,01	0,844	0,751	0,037	0,037	-0,081	–	0,064	-0,084	–	0,061
**Right fusiform gyrus**	-0,01	-0,01	0,864	0,846	0,037	0,037	-0,080	–	0,066	-0,080	–	0,066
**Left rostral anterior cingulate cortex**	-0,01	-0,01	0,870	0,820	0,037	0,037	-0,080	–	0,066	-0,081	–	0,065
**Left medial orbitofrontal cortex**	0,00	-0,01	0,934	0,864	0,037	0,037	-0,076	–	0,069	-0,079	–	0,066
**Left rostral middle frontal gyrus**	0,00	0,00	0,954	0,918	0,037	0,037	-0,076	–	0,070	-0,076	–	0,069

**Left temporal pole**	0,00	0,00	0,969	0,935	0,037	0,037	-0,074	–	0,071	-0,075	–	0,069
**Right pars orbitalis**	0,00	0,00	0,992	0,974	0,037	0,037	-0,073	–	0,072	-0,074	–	0,071
**Left precentral gyrus**	0,00	0,00	0,985	0,944	0,037	0,037	-0,073	–	0,073	-0,076	–	0,071
**Left middle temporal gyrus**	0,01	0,00	0,876	0,900	0,039	0,039	-0,071	–	0,081	-0,071	–	0,081
**Right lateral orbitofrontal cortex**	0,01	0,01	0,829	0,860	0,037	0,037	-0,065	–	0,080	-0,066	–	0,079
**Right rostral middle frontal gyrus**	0,01	0,01	0,788	0,823	0,037	0,037	-0,063	–	0,082	-0,064	–	0,081
**Left caudal anterior cingulate cortex**	0,02	0,01	0,674	0,715	0,037	0,037	-0,058	–	0,088	-0,059	–	0,087
**Right frontal pole**	0,02	0,01	0,661	0,715	0,037	0,037	-0,057	–	0,088	-0,059	–	0,086
**Right parahippocampal gyrus**	0,02	0,01	0,648	0,681	0,037	0,037	-0,056	–	0,090	-0,057	–	0,088
**Right inferior temporal gyrus**	0,02	0,01	0,634	0,660	0,037	0,037	-0,055	–	0,091	-0,056	–	0,089
**Right temporal pole**	0,02	0,01	0,575	0,710	0,037	0,037	-0,052	–	0,094	-0,059	–	0,087
**Left banks superior temporal sulcus**	0,02	0,02	0,591	0,614	0,039	0,039	-0,056	–	0,097	-0,057	–	0,096
**Right banks superior temporal sulcus**	0,03	0,02	0,506	0,528	0,038	0,038	-0,050	–	0,101	-0,051	–	0,100
**Right paracentral lobule**	0,03	0,02	0,480	0,519	0,037	0,037	-0,048	–	0,098	-0,048	–	0,097
**Left fusiform gyrus**	0,03	0,02	0,472	0,511	0,037	0,037	-0,046	–	0,099	-0,048	–	0,097
**Right medial orbitofrontal cortex**	0,03	0,02	0,460	0,497	0,037	0,037	-0,046	–	0,099	-0,047	–	0,098
**Left precuneus cortex**	0,03	0,03	0,374	0,399	0,037	0,037	-0,040	–	0,105	-0,041	–	0,104
**Left inferior parietal cortex**	0,04	0,03	0,355	0,390	0,038	0,038	-0,039	–	0,110	-0,041	–	0,107
**Left parahippocampal gyrus**	0,05	0,05	0,177	0,196	0,037	0,037	-0,022	–	0,124	-0,024	–	0,121
**Right inferior parietal cortex**	0,05	0,05	0,155	0,170	0,038	0,038	-0,020	–	0,128	-0,022	–	0,126

Abbreviations: LR = linear regression; LMEri= linear mixed-effects random-intercept model

Supplementary table S7: Full mega-analytic results for cortical thickness of each structure for the pediatric OCD patients compared with pediatric healthy controls, controlling for age, sex and scan center.

	Cohen's d		P-value		Standard Error		95% Confidence Interval					
	LR	LMEri	LR	LMEri	LR	LMEri	LR			LMEri		
**Left inferior parietal cortex**	**-0,31**	**-0,28**	**<0,001**	**<0,001**	**0,077**	**0,077**	**-0,457**	**–**	**-0,154**	**-0,455**	**–**	**-0,151**
**Right superior parietal cortex**	**-0,27**	**-0,21**	**<0,001**	**<0,001**	**0,075**	**0,075**	**-0,416**	**–**	**-0,121**	**-0,415**	**–**	**-0,119**
**Left lateral occipital cortex**	**-0,26**	**-0,23**	**<0,001**	**<0,001**	**0,075**	**0,075**	**-0,404**	**–**	**-0,109**	**-0,401**	**–**	**-0,106**
**Left superior parietal cortex**	-0,24	-0,20	0,001	0,002	0,075	0,075	-0,389	–	-0,093	-0,388	–	-0,093
**Left precuneus cortex**	-0,21	-0,19	0,006	0,007	0,075	0,075	-0,354	–	-0,060	-0,352	–	-0,058
**Left cuneus cortex**	-0,18	-0,17	0,015	0,018	0,075	0,075	-0,331	–	-0,037	-0,327	–	-0,032
**Right inferior parietal cortex**	-0,17	-0,17	0,003	0,004	0,076	0,076	-0,374	–	-0,075	-0,373	–	-0,074
**Right lateral occipital cortex**	-0,16	-0,13	0,031	0,034	0,075	0,075	-0,310	–	-0,016	-0,308	–	-0,014
**Left thickness**	-0,16	-0,14	0,033	0,034	0,075	0,075	-0,307	–	-0,015	-0,305	–	-0,013
**Right cuneus cortex**	-0,16	-0,15	0,038	0,039	0,075	0,075	-0,303	–	-0,010	-0,303	–	-0,009
**Right precuneus cortex**	-0,15	-0,14	0,044	0,048	0,075	0,075	-0,299	–	-0,006	-0,296	–	-0,002
**Left supramarginal gyrus**	-0,15	-0,13	0,052	0,055	0,077	0,077	-0,303	–	0,000	-0,301	–	0,002
**Right thickness**	-0,14	-0,13	0,055	0,057	0,075	0,075	-0,291	–	0,002	-0,289	–	0,003
**Left pericalcarine cortex**	-0,14	-0,14	0,059	0,057	0,075	0,075	-0,290	–	0,004	-0,291	–	0,004
**Right caudal middle frontal gyrus**	-0,13	-0,11	0,079	0,079	0,075	0,075	-0,281	–	0,014	-0,281	–	0,014
**Left posterior cingulate cortex**	-0,13	-0,13	0,082	0,077	0,075	0,075	-0,279	–	0,015	-0,280	–	0,014
**Right pericalcarine cortex**	-0,13	-0,13	0,085	0,081	0,075	0,075	-0,277	–	0,017	-0,278	–	0,015
**Left banks superior temporal sulcus**	-0,13	-0,12	0,099	0,115	0,078	0,078	-0,283	–	0,023	-0,277	–	0,029
**Left isthmus cingulate cortex**	-0,12	-0,12	0,100	0,087	0,075	0,075	-0,271	–	0,023	-0,276	–	0,018
**Right banks superior temporal sulcus**	-0,11	-0,10	0,158	0,163	0,077	0,077	-0,262	–	0,041	-0,260	–	0,043
**Left paracentral lobule**	-0,11	-0,10	0,147	0,155	0,075	0,075	-0,256	–	0,037	-0,254	–	0,039
**Right posterior cingulate cortex**	-0,10	-0,10	0,183	0,170	0,075	0,075	-0,247	–	0,046	-0,250	–	0,043
**Left precentral gyrus**	-0,10	-0,08	0,194	0,200	0,076	0,076	-0,247	–	0,049	-0,246	–	0,050
**Left transverse temporal cortex**	-0,10	-0,08	0,193	0,189	0,075	0,075	-0,245	–	0,048	-0,245	–	0,048
**Right postcentral gyrus**	-0,10	-0,08	0,213	0,225	0,076	0,076	-0,245	–	0,053	-0,242	–	0,056
**Left fusiform gyrus**	-0,09	-0,07	0,229	0,233	0,075	0,075	-0,240	–	0,056	-0,239	–	0,057
**Right insula**	-0,09	-0,08	0,248	0,262	0,076	0,076	-0,238	–	0,060	-0,235	–	0,063
**Left postcentral gyrus**	-0,09	-0,07	0,267	0,280	0,076	0,076	-0,234	–	0,064	-0,232	–	0,066
**Left inferior temporal gyrus**	-0,09	-0,07	0,265	0,269	0,075	0,075	-0,233	–	0,063	-0,232	–	0,064
**Right inferior temporal gyrus**	-0,08	-0,06	0,275	0,288	0,075	0,075	-0,231	–	0,064	-0,228	–	0,067
**Left middle temporal gyrus**	-0,08	-0,07	0,282	0,294	0,076	0,076	-0,232	–	0,067	-0,230	–	0,069
**Right precentral gyrus**	-0,08	-0,07	0,300	0,296	0,076	0,076	-0,228	–	0,069	-0,228	–	0,069
**Right middle temporal gyrus**	-0,08	-0,06	0,314	0,314	0,076	0,076	-0,227	–	0,072	-0,227	–	0,072
**Right rostral middle frontal gyrus**	-0,08	-0,07	0,319	0,290	0,075	0,075	-0,223	–	0,072	-0,227	–	0,067
**Left medial orbitofrontal cortex**	-0,07	-0,07	0,325	0,309	0,075	0,075	-0,222	–	0,072	-0,224	–	0,070
**Right supramarginal gyrus**	-0,07	-0,06	0,346	0,358	0,077	0,077	-0,224	–	0,077	-0,222	–	0,079
**Right rostral anterior cingulate cortex**	-0,07	-0,07	0,346	0,314	0,076	0,076	-0,221	–	0,076	-0,225	–	0,072
**Right paracentral lobule**	-0,07	-0,06	0,347	0,365	0,075	0,075	-0,217	–	0,076	-0,215	–	0,078
**Right parahippocampal gyrus**	-0,07	-0,06	0,371	0,358	0,075	0,075	-0,216	–	0,080	-0,218	–	0,078
**Left lingual gyrus**	-0,07	-0,06	0,370	0,376	0,075	0,075	-0,214	–	0,079	-0,214	–	0,080
**Right fusiform gyrus**	-0,07	-0,05	0,377	0,385	0,076	0,076	-0,215	–	0,081	-0,214	–	0,082
**Left pars opercularis**	-0,06	-0,05	0,404	0,424	0,075	0,075	-0,211	–	0,084	-0,208	–	0,087
**Right superior frontal gyrus**	-0,06	-0,06	0,430	0,402	0,076	0,076	-0,209	–	0,088	-0,213	–	0,085
**Left superior frontal gyrus**	-0,06	-0,05	0,427	0,425	0,075	0,075	-0,208	–	0,087	-0,208	–	0,087
**Left caudal middle frontal gyrus**	-0,06	-0,04	0,428	0,436	0,075	0,075	-0,206	–	0,087	-0,205	–	0,088
**Right pars triangularis**	-0,06	-0,06	0,457	0,442	0,075	0,075	-0,203	–	0,090	-0,205	–	0,089
**Right pars orbitalis**	-0,05	-0,05	0,537	0,518	0,075	0,075	-0,193	–	0,100	-0,195	–	0,098
**Left entorhinal cortex**	-0,05	-0,04	0,571	0,560	0,079	0,079	-0,199	–	0,109	-0,200	–	0,108
**Left frontal pole**	-0,04	-0,05	0,556	0,523	0,075	0,075	-0,191	–	0,102	-0,194	–	0,099
**Right pars opercularis**	-0,04	-0,04	0,610	0,606	0,075	0,075	-0,186	–	0,109	-0,187	–	0,108

**Left parahippocampal gyrus**	-0,04	-0,03	0,611	0,605	0,075	0,075	-0,187	–	0,109	-0,187	–	0,109
**Right superior temporal gyrus**	-0,04	-0,03	0,624	0,624	0,077	0,077	-0,189	–	0,113	-0,189	–	0,113
**Right isthmus cingulate cortex**	-0,04	-0,04	0,631	0,588	0,075	0,075	-0,183	–	0,110	-0,187	–	0,106
**Left rostral middle frontal gyrus**	-0,03	-0,02	0,731	0,730	0,075	0,075	-0,172	–	0,120	-0,172	–	0,120
**Right transverse temporal cortex**	-0,02	-0,02	0,767	0,765	0,075	0,075	-0,169	–	0,124	-0,169	–	0,124
**Right entorhinal cortex**	-0,02	-0,01	0,817	0,824	0,081	0,081	-0,177	–	0,139	-0,176	–	0,140
**Left pars orbitalis**	-0,02	-0,02	0,824	0,795	0,075	0,075	-0,163	–	0,130	-0,166	–	0,127
**Left pars triangularis**	-0,01	-0,01	0,906	0,895	0,075	0,075	-0,156	–	0,138	-0,157	–	0,137
**Left insula**	0,00	0,00	0,953	0,974	0,076	0,076	-0,153	–	0,144	-0,151	–	0,146
**Left caudal anterior cingulate cortex**	0,00	0,00	0,983	0,978	0,075	0,075	-0,146	–	0,149	-0,150	–	0,146
**Right lingual gyrus**	0,01	0,01	0,936	0,931	0,075	0,075	-0,140	–	0,152	-0,140	–	0,153
**Left superior temporal gyrus**	0,01	0,01	0,873	0,864	0,077	0,077	-0,138	–	0,163	-0,138	–	0,164
**Right temporal pole**	0,02	0,02	0,778	0,778	0,075	0,075	-0,126	–	0,168	-0,126	–	0,168
**Left temporal pole**	0,03	0,02	0,706	0,708	0,075	0,075	-0,118	–	0,176	-0,119	–	0,175
**Right lateral orbitofrontal cortex**	0,04	0,04	0,597	0,588	0,075	0,075	-0,106	–	0,186	-0,106	–	0,187
**Right frontal pole**	0,04	0,03	0,576	0,623	0,075	0,075	-0,104	–	0,189	-0,109	–	0,184
**Right caudal anterior cingulate cortex**	0,06	0,05	0,397	0,429	0,076	0,076	-0,084	–	0,213	-0,088	–	0,209
**Left rostral anterior cingulate cortex**	0,08	0,07	0,315	0,331	0,076	0,075	-0,071	–	0,225	-0,074	–	0,222
**Right medial orbitofrontal cortex**	0,10	0,08	0,208	0,212	0,075	0,075	-0,052	–	0,243	-0,053	–	0,242
**Left lateral orbitofrontal cortex**	0,10	0,09	0,190	0,188	0,075	0,075	-0,047	–	0,245	-0,047	–	0,245

Abbreviations: LR = linear regression; LMEri= linear mixed-effects random-intercept model

Supplementary table S8: Full mega-analytic results for cortical surface area of each structure for the pediatric OCD patients compared with pediatric healthy controls, controlling for age, sex, scan center, and ICV.

	Cohen's d		P-value		Standard Error		95% Confidence Interval					
	LR	LMEri	LR	LMEri	LR	LMEri	LR			LMEri		
**Right medial orbitofrontal cortex**	-0,25	-0,24	0,001	0,001	0,076	0,075	-0,400	–	-0,104	-0,401	–	-0,105
**Left posterior cingulate cortex**	-0,23	-0,23	0,002	0,002	0,075	0,075	-0,378	–	-0,083	-0,381	–	-0,086
**Right paracentral lobule**	-0,22	-0,22	0,003	0,002	0,075	0,075	-0,370	–	-0,076	-0,376	–	-0,082
**Left medial orbitofrontal cortex**	-0,19	-0,20	0,013	0,009	0,075	0,075	-0,335	–	-0,041	-0,345	–	-0,051
**Left inferior parietal cortex**	-0,19	-0,18	0,016	0,018	0,077	0,077	-0,339	–	-0,037	-0,335	–	-0,033
**Right superior frontal gyrus**	-0,18	-0,18	0,016	0,014	0,076	0,076	-0,334	–	-0,036	-0,336	–	-0,038
**Left lateral orbitofrontal cortex**	-0,18	-0,18	0,018	0,014	0,075	0,075	-0,325	–	-0,032	-0,331	–	-0,038
**Left paracentral lobule**	-0,17	-0,17	0,024	0,019	0,075	0,075	-0,318	–	-0,024	-0,323	–	-0,029
**Left rostral middle frontal gyrus**	-0,17	-0,16	0,026	0,024	0,075	0,075	-0,315	–	-0,022	-0,317	–	-0,023
**Right posterior cingulate cortex**	-0,17	-0,16	0,028	0,030	0,075	0,075	-0,313	–	-0,019	-0,311	–	-0,017
**Right rostral middle frontal gyrus**	-0,15	-0,14	0,042	0,040	0,075	0,075	-0,302	–	-0,007	-0,302	–	-0,008
**Left superior frontal gyrus**	-0,15	-0,14	0,057	0,050	0,076	0,075	-0,293	–	0,003	-0,297	–	-0,001
**Right fusiform gyrus**	-0,13	-0,13	0,084	0,089	0,076	0,076	-0,280	–	0,016	-0,278	–	0,018
**Left postcentral gyrus**	-0,13	-0,12	0,092	0,092	0,076	0,076	-0,278	–	0,020	-0,278	–	0,020
**Right cuneus cortex**	-0,13	-0,13	0,096	0,072	0,075	0,075	-0,273	–	0,021	-0,282	–	0,012
**Left rostral anterior cingulate cortex**	-0,12	-0,11	0,122	0,115	0,076	0,076	-0,266	–	0,030	-0,268	–	0,028
**Left caudal anterior cingulate cortex**	-0,12	-0,12	0,122	0,109	0,076	0,076	-0,266	–	0,030	-0,270	–	0,026
**Right surface area**	-0,11	-0,10	0,128	0,120	0,075	0,075	-0,261	–	0,031	-0,263	–	0,029
**Right pars orbitalis**	-0,11	-0,12	0,129	0,097	0,075	0,075	-0,261	–	0,032	-0,271	–	0,022
**Left pars orbitalis**	-0,11	-0,12	0,133	0,096	0,075	0,075	-0,260	–	0,033	-0,271	–	0,022
**Left insula**	-0,11	-0,11	0,162	0,146	0,076	0,076	-0,256	–	0,041	-0,260	–	0,038
**Right lateral occipital cortex**	-0,10	-0,10	0,177	0,156	0,075	0,075	-0,249	–	0,045	-0,254	–	0,040
**Left middle temporal gyrus**	-0,10	-0,10	0,187	0,185	0,076	0,076	-0,251	–	0,048	-0,251	–	0,048
**Right lateral orbitofrontal cortex**	-0,10	-0,10	0,188	0,160	0,075	0,075	-0,246	–	0,047	-0,252	–	0,041
**Right pars triangularis**	-0,10	-0,10	0,192	0,171	0,075	0,075	-0,246	–	0,048	-0,250	–	0,044
**Left surface area**	-0,10	-0,09	0,198	0,187	0,075	0,074	-0,243	–	0,049	-0,245	–	0,047
**Left cuneus cortex**	-0,10	-0,09	0,210	0,207	0,075	0,075	-0,242	–	0,052	-0,242	–	0,051
**Left pericalcarine cortex**	-0,09	-0,09	0,216	0,216	0,075	0,075	-0,241	–	0,053	-0,241	–	0,053
**Right superior parietal cortex**	-0,08	-0,08	0,275	0,265	0,075	0,075	-0,230	–	0,064	-0,231	–	0,063
**Left precuneus cortex**	-0,08	-0,08	0,293	0,289	0,075	0,075	-0,227	–	0,067	-0,227	–	0,067
**Right inferior parietal cortex**	-0,07	-0,06	0,352	0,373	0,076	0,076	-0,221	–	0,078	-0,218	–	0,080
**Left superior parietal cortex**	-0,07	-0,06	0,391	0,396	0,075	0,075	-0,213	–	0,082	-0,212	–	0,083
**Left pars opercularis**	-0,06	-0,06	0,393	0,419	0,075	0,075	-0,212	–	0,082	-0,209	–	0,086
**Right lingual gyrus**	-0,06	-0,06	0,398	0,448	0,075	0,075	-0,210	–	0,082	-0,204	–	0,089
**Right pericalcarine cortex**	-0,05	-0,05	0,496	0,480	0,075	0,075	-0,198	–	0,095	-0,200	–	0,093
**Right parahippocampal gyrus**	-0,05	-0,03	0,552	0,643	0,076	0,075	-0,193	–	0,103	-0,184	–	0,112
**Right postcentral gyrus**	-0,04	-0,04	0,585	0,621	0,076	0,076	-0,191	–	0,107	-0,187	–	0,111
**Left lingual gyrus**	-0,04	-0,03	0,600	0,655	0,075	0,075	-0,186	–	0,107	-0,181	–	0,112
**Right middle temporal gyrus**	-0,04	-0,04	0,618	0,606	0,076	0,076	-0,188	–	0,111	-0,189	–	0,110
**Right pars opercularis**	-0,03	-0,04	0,653	0,626	0,075	0,075	-0,182	–	0,113	-0,184	–	0,111
**Right superior temporal gyrus**	-0,03	-0,03	0,663	0,662	0,077	0,077	-0,185	–	0,117	-0,185	–	0,117
**Right supramarginal gyrus**	-0,03	-0,03	0,672	0,700	0,077	0,077	-0,183	–	0,118	-0,180	–	0,120
**Right insula**	-0,03	-0,03	0,685	0,637	0,076	0,076	-0,180	–	0,118	-0,185	–	0,113
**Left banks superior temporal sulcus**	-0,03	-0,03	0,711	0,710	0,078	0,078	-0,182	–	0,124	-0,182	–	0,123
**Left precentral gyrus**	-0,03	-0,03	0,704	0,699	0,076	0,076	-0,177	–	0,119	-0,178	–	0,119
**Left fusiform gyrus**	-0,03	-0,03	0,722	0,671	0,075	0,075	-0,175	–	0,121	-0,180	–	0,116
**Left caudal middle frontal gyrus**	-0,02	-0,02	0,832	0,763	0,075	0,075	-0,163	–	0,131	-0,169	–	0,124
**Left frontal pole**	-0,01	-0,02	0,857	0,828	0,075	0,075	-0,160	–	0,133	-0,162	–	0,130
**Right precuneus cortex**	-0,01	-0,01	0,924	0,898	0,075	0,075	-0,154	–	0,139	-0,156	–	0,137
**Left superior temporal gyrus**	-0,01	-0,01	0,943	0,927	0,077	0,077	-0,156	–	0,145	-0,158	–	0,144

**Right caudal anterior cingulate cortex**	0,00	-0,01	0,977	0,946	0,076	0,076	-0,150	–	0,146	-0,153	–	0,143
**Right temporal pole**	0,01	0,03	0,896	0,715	0,075	0,075	-0,137	–	0,157	-0,121	–	0,173
**Right rostral anterior cingulate cortex**	0,01	0,01	0,891	0,937	0,076	0,076	-0,138	–	0,159	-0,142	–	0,155
**Left lateral occipital cortex**	0,01	0,01	0,844	0,888	0,075	0,075	-0,132	–	0,162	-0,136	–	0,158
**Left entorhinal cortex**	0,02	0,03	0,827	0,724	0,079	0,079	-0,137	–	0,172	-0,127	–	0,181
**Right inferior temporal gyrus**	0,02	0,02	0,819	0,823	0,075	0,075	-0,130	–	0,165	-0,131	–	0,164
**Right precentral gyrus**	0,02	0,02	0,819	0,812	0,076	0,076	-0,131	–	0,166	-0,130	–	0,167
**Right caudal middle frontal gyrus**	0,02	0,01	0,799	0,855	0,075	0,075	-0,128	–	0,167	-0,133	–	0,162
**Left parahippocampal gyrus**	0,02	0,03	0,769	0,663	0,075	0,075	-0,125	–	0,170	-0,115	–	0,180
**Right banks superior temporal sulcus**	0,03	0,02	0,731	0,737	0,077	0,077	-0,125	–	0,178	-0,125	–	0,178
**Left transverse temporal cortex**	0,03	0,04	0,664	0,627	0,075	0,075	-0,114	–	0,179	-0,110	–	0,183
**Right isthmus cingulate cortex**	0,04	0,04	0,602	0,627	0,075	0,075	-0,107	–	0,186	-0,110	–	0,184
**Left inferior temporal gyrus**	0,04	0,04	0,601	0,631	0,075	0,075	-0,108	–	0,188	-0,111	–	0,185
**Left pars triangularis**	0,04	0,04	0,596	0,611	0,075	0,075	-0,107	–	0,187	-0,108	–	0,185
**Right transverse temporal cortex**	0,05	0,04	0,527	0,536	0,075	0,075	-0,099	–	0,194	-0,100	–	0,193
**Left supramarginal gyrus**	0,05	0,05	0,497	0,535	0,077	0,077	-0,098	–	0,204	-0,103	–	0,200
**Right entorhinal cortex**	0,08	0,08	0,352	0,295	0,081	0,081	-0,082	–	0,234	-0,073	–	0,243
**Left isthmus cingulate cortex**	0,13	0,13	0,089	0,090	0,075	0,075	-0,018	–	0,276	-0,019	–	0,275
**Left temporal pole**	0,13	0,12	0,084	0,098	0,075	0,075	-0,016	–	0,278	-0,021	–	0,273
**Right frontal pole**	0,13	0,12	0,083	0,097	0,075	0,075	-0,016	–	0,278	-0,021	–	0,273

Abbreviations: LR = linear regression; LMEri= linear mixed-effects random-intercept model

Supplementary table S9: Comparisons of goodness-of-fit between the different mega-analysis cortical thickness models of the adult sample.

	BIC LR	BIC LMEri
Left banks superior temporal sulcus	10512	10454
Left caudal anterior cingulate cortex	14465	14384
Left caudal middle frontal gyrus	10852	10804
Left cuneus cortex	10031	9969
Left entorhinal cortex	14898	14821
Left frontal pole	15099	15022
Left fusiform gyrus	10632	10584
Left inferior parietal cortex	9693	9638
Left inferior temporal gyrus	11059	11001
Left insula	11454	11391
Left isthmus cingulate cortex	12636	12559
Left lateral occipital cortex	10172	10122
Left lateral orbitofrontal cortex	11437	11367
Left lingual gyrus	9969	9906
Left medial orbitofrontal cortex	11734	11665
Left middle temporal gyrus	10392	10332
Left paracentral lobule	10984	10940
Left parahippocampal gyrus	14977	14903
Left pars opercularis	10735	10676
Left pars orbitalis	13067	12989
Left pars triangularis	11248	11178
Left pericalcarine cortex	9938	9881
Left postcentral gyrus	9220	9174
Left posterior cingulate cortex	11218	11153
Left precentral gyrus	10015	9988
Left precuneus cortex	10167	10106
Left rostral anterior cingulate cortex	13631	13554
Left rostral middle frontal gyrus	10282	10234
Left superior frontal gyrus	10662	10616
Left superior parietal cortex	9400	9351
Left superior temporal gyrus	10148	10092
Left supramarginal gyrus	9574	9521
Left temporal pole	15902	15818
Left thickness	8403	8358
Left transverse temporal cortex	12964	12901
Right banks superior temporal sulcus	10912	10851
Right caudal anterior cingulate cortex	13772	13693
Right caudal middle frontal gyrus	10995	10940
Right cuneus cortex	10150	10095
Right entorhinal cortex	14518	14448
Right frontal pole	15119	15050
Right fusiform gyrus	10506	10465
Right inferior parietal cortex	9799	9750

**Right inferior temporal gyrus**	11206	11152
**Right insula**	11692	11633
**Right isthmus cingulate cortex**	12432	12362
**Right lateral occipital cortex**	10209	10162
**Right lateral orbitofrontal cortex**	11641	11587
**Right lingual gyrus**	9909	9858
**Right medial orbitofrontal cortex**	12014	11945
**Right middle temporal gyrus**	10831	10778
**Right paracentral lobule**	10933	10896
**Right parahippocampal gyrus**	13924	13860
**Right pars opercularis**	11050	10987
**Right pars orbitalis**	13085	13017
**Right pars triangularis**	11044	10973
**Right pericalcarine cortex**	9936	9877
**Right postcentral gyrus**	9406	9359
**Right posterior cingulate cortex**	11343	11280
**Right precentral gyrus**	10086	10054
**Right precuneus cortex**	10216	10167
**Right rostral anterior cingulate cortex**	13509	13437
**Right rostral middle frontal gyrus**	10192	10136
**Right superior frontal gyrus**	10648	10593
**Right superior parietal cortex**	9534	9487
**Right superior temporal gyrus**	10308	10255
**Right supramarginal gyrus**	9691	9635
**Right temporal pole**	16069	16009
**Right thickness**	8415	8370
**Right transverse temporal cortex**	13006	12941

Abbreviations: LR = linear regression; LMEri= linear mixed-effects random-intercept model; BIC = Bayesian information criterion

Supplementary table S10: Comparisons of goodness-of-fit between the different mega-analysis cortical surface area models of the adult sample.

	BIC LR	BIC LMEri
**Left banks superior temporal sulcus**	33917	33822
**Left caudal anterior cingulate cortex**	36033	35927
**Left caudal middle frontal gyrus**	41892	41792
**Left cuneus cortex**	38021	37939
**Left entorhinal cortex**	30926	30823
**Left frontal pole**	28840	28743
**Left fusiform gyrus**	42487	42394
**Left inferior parietal cortex**	42943	42859
**Left inferior temporal gyrus**	42148	42063
**Left insula**	38673	38602
**Left isthmus cingulate cortex**	37647	37548
**Left lateral occipital cortex**	43927	43830
**Left lateral orbitofrontal cortex**	40233	40157
**Left lingual gyrus**	42655	42575
**Left medial orbitofrontal cortex**	39350	39253
**Left middle temporal gyrus**	38588	38510
**Left paracentral lobule**	38051	37965
**Left parahippocampal gyrus**	34924	34825
**Left pars opercularis**	40449	40346
**Left pars orbitalis**	33325	33232
**Left pars triangularis**	38619	38509
**Left pericalcarine cortex**	38914	38837
**Left postcentral gyrus**	42437	42346
**Left posterior cingulate cortex**	37722	37630
**Left precentral gyrus**	42785	42694
**Left precuneus cortex**	42560	42482
**Left rostral anterior cingulate cortex**	36400	36317
**Left rostral middle frontal gyrus**	45199	45124
**Left superior frontal gyrus**	44387	44311
**Left superior parietal cortex**	44394	44296
**Left superior temporal gyrus**	38176	38086
**Left supramarginal gyrus**	40260	40170
**Left surface area**	57918	57854
**Left temporal pole**	32195	32104
**Left transverse temporal cortex**	33368	33263
**Right banks superior temporal sulcus**	33860	33771
**Right caudal anterior cingulate cortex**	37094	36982
**Right caudal middle frontal gyrus**	41998	41897
**Right cuneus cortex**	37872	37790
**Right entorhinal cortex**	31878	31780
**Right frontal pole**	30280	30179

**Right fusiform gyrus**	41836	41748
**Right inferior parietal cortex**	44103	44014
**Right inferior temporal gyrus**	42434	42352
**Right insula**	39089	39019
**Right isthmus cingulate cortex**	37017	36928
**Right lateral occipital cortex**	44112	44015
**Right lateral orbitofrontal cortex**	40732	40660
**Right lingual gyrus**	42475	42402
**Right medial orbitofrontal cortex**	38206	38127
**Right middle temporal gyrus**	41332	41253
**Right paracentral lobule**	39026	38939
**Right parahippocampal gyrus**	34564	34465
**Right pars opercularis**	39229	39119
**Right pars orbitalis**	34661	34562
**Right pars triangularis**	39321	39217
**Right pericalcarine cortex**	39265	39199
**Right postcentral gyrus**	42532	42441
**Right posterior cingulate cortex**	37973	37888
**Right precentral gyrus**	43015	42918
**Right precuneus cortex**	43123	43041
**Right rostral anterior cingulate cortex**	35884	35785
**Right rostral middle frontal gyrus**	45383	45303
**Right superior frontal gyrus**	45077	44997
**Right superior parietal cortex**	44124	44024
**Right superior temporal gyrus**	38427	38337
**Right supramarginal gyrus**	40484	40392
**Right surface area**	57957	57891
**Right temporal pole**	31878	31750
**Right transverse temporal cortex**	31641	31543

Abbreviations: LR = linear regression; LMEri= linear mixed-effects random-intercept model; BIC = Bayesian information criterion

Supplementary table S11: Comparisons of goodness-of-fit between the different mega-analysis cortical thickness models of the pediatric sample.

	BIC LR	BIC LMEri
**Left banks superior temporal sulcus**	2902	2869
**Left caudal anterior cingulate cortex**	3606	3587
**Left caudal middle frontal gyrus**	2971	2966
**Left cuneus cortex**	2688	2655
**Left entorhinal cortex**	3668	3656
**Left frontal pole**	4061	4028
**Left fusiform gyrus**	2635	2625
**Left inferior parietal cortex**	2634	2610
**Left inferior temporal gyrus**	2978	2962
**Left insula**	2847	2824
**Left isthmus cingulate cortex**	3296	3267
**Left lateral occipital cortex**	2464	2444
**Left lateral orbitofrontal cortex**	3095	3077
**Left lingual gyrus**	2609	2583
**Left medial orbitofrontal cortex**	3130	3104
**Left middle temporal gyrus**	2969	2948
**Left paracentral lobule**	2989	2969
**Left parahippocampal gyrus**	3714	3694
**Left pars opercularis**	2878	2857
**Left pars orbitalis**	3549	3517
**Left pars triangularis**	3034	3010
**Left pericalcarine cortex**	2716	2684
**Left postcentral gyrus**	2418	2403
**Left posterior cingulate cortex**	3016	2989
**Left precentral gyrus**	2660	2650
**Left precuneus cortex**	2734	2711
**Left rostral anterior cingulate cortex**	3558	3539
**Left rostral middle frontal gyrus**	2791	2776
**Left superior frontal gyrus**	2908	2898
**Left superior parietal cortex**	2599	2585
**Left superior temporal gyrus**	2825	2813
**Left supramarginal gyrus**	2654	2637
**Left temporal pole**	4219	4207
**Left thickness**	2245	2227
**Left transverse temporal cortex**	3359	3343
**Right banks superior temporal sulcus**	3007	2983
**Right caudal anterior cingulate cortex**	3539	3522
**Right caudal middle frontal gyrus**	3033	3017
**Right cuneus cortex**	2839	2804
**Right entorhinal cortex**	3511	3500
**Right frontal pole**	4017	3993
**Right fusiform gyrus**	2676	2665
**Right inferior parietal cortex**	2744	2735

**Right inferior temporal gyrus**	2948	2937
**Right insula**	2966	2947
**Right isthmus cingulate cortex**	3282	3252
**Right lateral occipital cortex**	2543	2532
**Right lateral orbitofrontal cortex**	3051	3028
**Right lingual gyrus**	2666	2643
**Right medial orbitofrontal cortex**	3164	3149
**Right middle temporal gyrus**	2922	2911
**Right paracentral lobule**	2978	2957
**Right parahippocampal gyrus**	3578	3559
**Right pars opercularis**	2983	2953
**Right pars orbitalis**	3478	3447
**Right pars triangularis**	3020	2991
**Right pericalcarine cortex**	2734	2706
**Right postcentral gyrus**	2484	2466
**Right posterior cingulate cortex**	2914	2890
**Right precentral gyrus**	2683	2666
**Right precuneus cortex**	2704	2681
**Right rostral anterior cingulate cortex**	3584	3566
**Right rostral middle frontal gyrus**	2817	2797
**Right superior frontal gyrus**	2908	2890
**Right superior parietal cortex**	2645	2634
**Right superior temporal gyrus**	2817	2809
**Right supramarginal gyrus**	2646	2633
**Right temporal pole**	4253	4243
**Right thickness**	2263	2242
**Right transverse temporal cortex**	3374	3360

Abbreviations: LR = linear regression; LMEri= linear mixed-effects random-intercept model; BIC = Bayesian information criterion

Supplementary table S12: Comparisons of goodness-of-fit between the different mega-analysis cortical surface area models of the pediatric sample.

	BIC LR	BIC LMEri
Left banks superior temporal sulcus	8777	8744
Left caudal anterior cingulate cortex	9027	8982
Left caudal middle frontal gyrus	10647	10607
Left cuneus cortex	9719	9679
Left entorhinal cortex	7618	7581
Left frontal pole	7362	7333
Left fusiform gyrus	10491	10461
Left inferior parietal cortex	10577	10545
Left inferior temporal gyrus	10740	10705
Left insula	9559	9532
Left isthmus cingulate cortex	9379	9341
Left lateral occipital cortex	11049	11022
Left lateral orbitofrontal cortex	10347	10319
Left lingual gyrus	10769	10722
Left medial orbitofrontal cortex	9881	9837
Left middle temporal gyrus	10105	10075
Left paracentral lobule	9599	9565
Left parahippocampal gyrus	8715	8672
Left pars opercularis	10034	10002
Left pars orbitalis	8478	8441
Left pars triangularis	9681	9649
Left pericalcarine cortex	9916	9871
Left postcentral gyrus	10519	10488
Left posterior cingulate cortex	9502	9465
Left precentral gyrus	10629	10607
Left precuneus cortex	10755	10726
Left rostral anterior cingulate cortex	9151	9121
Left rostral middle frontal gyrus	11476	11452
Left superior frontal gyrus	11241	11211
Left superior parietal cortex	11197	11163
Left superior temporal gyrus	10053	10023
Left supramarginal gyrus	10388	10348
Left surface area	14601	14581
Left temporal pole	8120	8081
Left transverse temporal cortex	8249	8211
Right banks superior temporal sulcus	8638	8613
Right caudal anterior cingulate cortex	9073	9040
Right caudal middle frontal gyrus	10469	10432
Right cuneus cortex	9817	9779
Right entorhinal cortex	7258	7223
Right frontal pole	7631	7596
Right fusiform gyrus	10402	10366
Right inferior parietal cortex	11048	11022

**Right inferior temporal gyrus**	10666	10633
**Right insula**	9572	9545
**Right isthmus cingulate cortex**	9194	9155
**Right lateral occipital cortex**	11110	11081
**Right lateral orbitofrontal cortex**	10337	10311
**Right lingual gyrus**	10833	10787
**Right medial orbitofrontal cortex**	9603	9575
**Right middle temporal gyrus**	10245	10219
**Right paracentral lobule**	9847	9813
**Right parahippocampal gyrus**	8631	8595
**Right pars opercularis**	9752	9714
**Right pars orbitalis**	8769	8735
**Right pars triangularis**	9959	9922
**Right pericalcarine cortex**	10033	9995
**Right postcentral gyrus**	10459	10426
**Right posterior cingulate cortex**	9472	9436
**Right precentral gyrus**	10558	10535
**Right precuneus cortex**	10847	10813
**Right rostral anterior cingulate cortex**	8875	8838
**Right rostral middle frontal gyrus**	11444	11422
**Right superior frontal gyrus**	11224	11198
**Right superior parietal cortex**	11223	11193
**Right superior temporal gyrus**	9948	9923
**Right supramarginal gyrus**	10494	10457
**Right surface area**	14627	14608
**Right temporal pole**	8056	8012
**Right transverse temporal cortex**	7991	7963

Abbreviations: LR = linear regression; LMEri= linear mixed-effects random-intercept model; BIC = Bayesian information criterion
